# Quantifying the Accuracy of Density Functionals on Transition Metal Bulk and Surface Properties

**DOI:** 10.1021/acs.jctc.3c00612

**Published:** 2023-11-09

**Authors:** David Vázquez-Parga, Andrea Fernández-Martínez, Francesc Viñes

**Affiliations:** Departament de Ciència de Materials i Química Física & Institut de Química Teòrica i Computacional (IQTCUB), Universitat de Barcelona, c/ Martí i Franquès 1, 08028 Barcelona, Spain

## Abstract

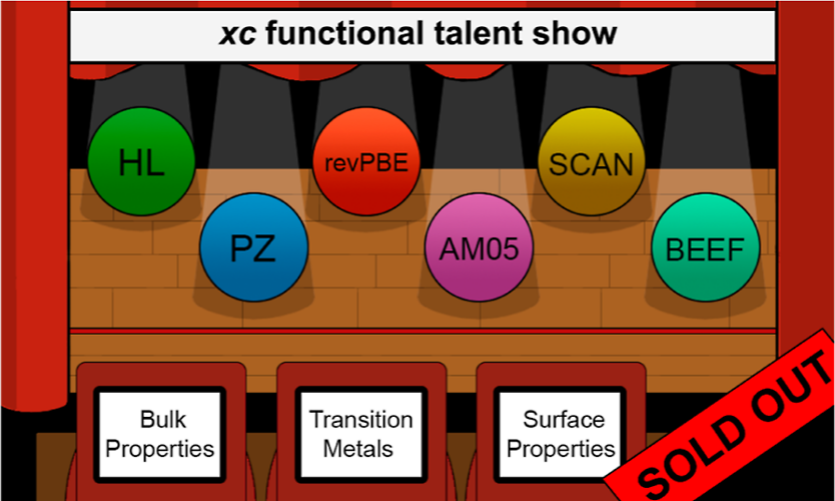

Density functional theory would be exact when the exact exchange–correlation (*xc*) functional would be known, but since it is regretfully not known, dozens of *xc* functionals have been developed in the past decades, with some of them better suited for describing certain systems and/or properties. For transition metals (TMs), recent systematic studies assessing bulk properties—shortest interatomic bond distance, δ, cohesive energy, *E*_coh_, and bulk modulus, *B*_0_—and surface features—surface energy, γ, work function, ϕ, and interlayer distances, δ_*ij*_—of 27 TM bulks and 81 TM surfaces, highlighted that generalized gradient approximation (GGA) based *xc* functionals are, overall, better suited than other types of *xc* functionals for the TMs bulk and surfaces description, such as Perdew–Burke–Ernzerhof (PBE) or Vega–Viñes (VV). Still, some basic local density approximation *xc* functionals were not assessed, such as the Hedin–Lundqvist (HL) and Perdew–Zunger (PZ), or GGAs such as the revised Perdew–Burke–Ernzerhof (revPBE) or the Armiento–Mattsson (AM05). Here, we expand the analysis by not only including them but also the recent meta-GGA strongly constrained appropriately normed (SCAN) *xc* functional, characterized by fulfilling all 17 mathematical conditions an *xc* must comply, plus the Bayesian error estimation functional (BEEF) *xc*, a functional parametrized over a large and diverse set of experimental results using machine learning. The present results reveal that none of the *xc* studied excel neither PBE nor VV, yet AM05 and SCAN performance is quite acceptable, while BEEF *xc* probably needs more shells of parametrization to reach competitive accuracy levels.

## 1 Introduction

During the last decade, density functional theory (DFT) has bloomed as the method of choice in describing diverse chemical systems, from molecules to solid-state materials, simply implying that a given chemical system energy is defined by its electron density function. However, even when DFT is theoretically well formulated, it still misses a key ingredient analytic formula, the so-called exchange–correlation (*xc*) functional, which has to be approximated. Since the very initial *xc* approaches within the local density approximation (LDA), dozens of functionals have appeared, mostly aimed at targeting a universal *xc* functional, which would allow describing accurately any type of system and property.

The *xc* functionals are customarily classified according to the Jacob’s Ladder of *xc* functional improvement, as posed by John P. Perdew,^1^ where the lowest rung is represented by LDA *xc* functionals, while higher rungs add accuracy and complexity up to the top, a heavenly region where the exact *xc* exists. Initial studies focused on adding rungs above LDA; for instance, while LDA *xc* functionals use only the electron density in their *ansätze*, the generalized gradient approximation (GGA) *xc* ones add the electron density gradient in them, while meta-GGAs also include the electron density second derivative. On a higher rung, hybrid functionals add a portion of Hartree–Fock exchange to the *xc* equation, and even higher rungs could be claimed, *e.g.*, by accounting for the exact exchange (EXX), analytically solved.^2^

So far, the collection of developed functionals fulfill Perdew’s dream, particularly as far as the thermochemistry of main group element molecules is concerned. However, most recent advances do not necessarily imply a better general description. For instance, it has been shown that the persistence of researchers on better describing the systems energetics caused a stray deviation from the path, impoverishing the description of the electron density.^3^ Furthermore, rising up the Jacob’s Ladder does not necessarily imply a better description, as seen, *e.g.*, on transition metals (TMs), where extensive studies on 30 TMs bulk and surface properties revealed GGA rung being better suited than more complex *xc* functionals,^4−6^ where the Perdew–Burke–Ernzerhof (PBE) *xc* functional^7^ has been found to be the most accurate over 15 different explored *xc* functionals from the first four rungs of Jacob’s Ladder for bulk properties,^4−6^ and its pole position remains when describing surface properties.^8^

These previous studies aimed at assessing the different DFT *xc* flavors when describing TM systems,^9^ but from a broader perspective, this is, evaluating different bulk and surface properties, at variance with the usual approach taken in *xc* development, where frequently only a single property is targeted, *i.e.*, the bulk shortest interatomic distance, δ, and normally considering few TM systems, typically late TMs, *e.g.*, Pd, Pt, Au, and so forth simply because such late TMs are the ones most studied and used as heterogeneous catalysts.^9^ As one could simply guess, the validation on a single property on a narrow data set may lead to large deviations and errors out of the evaluation data set, and this is exactly the rock-in-the-shoe for many *xc* functionals.^4−6,8^

Recent advances in the description of TM-based systems departed from PBE GGA and implied the adjustment of exchange and correlation coefficients, as in Vega–Viñes (VV) *xc* functional,^8^ or the recovery of the linear spin density (LSD) response in VV for solids (VVsol).^8^ As far as theoretical constrains are concerned, the strongly constrained and appropriately normed (SCAN) meta-GGA *xc* functional was recently developed so as to meet the 17 theoretical conditions that a meta-GGA *xc* must fulfil and was tested for different systems and properties.^10^ However, when it comes to solid bulks description, the evaluation was done only on the lattice constant parameter, taken from a previous set containing 20 solids, from which there were only four TMs; Cu, Rh, Pd, and Ag, and so, again being all late TMs, and all displaying a face-centered cubic (*fcc*) crystal structure.^10^ A question immediately arises here, whether such four TMs and a single property were enough to assess the *xc* accuracy for the full TM materials family, or whether deviations would arise when using earlier TMs, other bulk properties, or even TMs with different crystallographic structures.

Furthermore, newer *xc* functionals have been developed with the advent of artificial intelligence (AI). In this particular aspect, the Bayesian error estimation functional (BEEF) *xc* functional^11^ used machine learning (ML) algorithms to parametrize it to a plethora of experimental data, including datasets of molecular formation and reaction energies, molecular reaction barriers, non-covalent interactions such as van der Waals (vdW)—for what is called sometimes BEEF-vdW—, and bulks lattice constants, cohesive energies, and chemisorption energies on solid surfaces of 14 TMs. Therefore, one would expect a good overall description of BEEF, yet again body-centered cubic (*bcc*) and hexagonal close-packed (*hcp*) TM crystal structures were severely underrepresented, and so, a more complete evaluation should include them in the proof of the pudding as previously done for other *xc*.^4−6,8^

The present study mainly aims at evaluating the new SCAN and BEEF *xc* functionals in describing 27 TMs featuring *fcc*, *hcp*, and *bcc* crystal structures, by evaluating three different bulk properties: the shortest interatomic distance, δ; the cohesive energy, *E*_coh_; and the bulk modulus, *B*_0_, plus three different surface properties, including surface energies, γ; work functions, ϕ; and surface relaxations, Δ_*ij*_. Each surface property is evaluated for the three most stable surfaces of each TM,^12^ being (001), (011), and (111) for *bcc* and *fcc* TMs, and (0001), (101̅0), and (112̅0) for *hcp* TMs. Moreover, other earlier *xc* functionals often disregarded in the literature have been considered as well, including the LDA parameterization of Hedin–Lundqvist (HL)^13^ for the *xc* potential, and the Ceperley–Alder (CA)^14^ parameterization of the electron–gas correlation energy as done by Perdew–Zunger (PZ),^15^ where normally the Vosko–Wilk–Nusair (VWN)^16^ is often the functional of choice within LDA, and two GGA functionals, the Armiento–Mattsson (AM05), originally designed for the better description of surfaces, yet only tested on Pt,^17^ and the revised version of PBE (revPBE),^18^ argued to better describe the adsorption of atoms and molecules on solid surfaces.

## 2 Computational Details

The present DFT calculations have been carried out using the Vienna *ab initio* simulation package (VASP) suite.^19^ Core electrons were treated using projector augmented wave pseudopotentials.^20^ A plane-wave basis set for the valence electron density with a 415 eV cutoff for the kinetic energy was used. This ensures having energy estimates converged below the chemical accuracy of ∼0.04 eV. The electronic convergence criterion was always set to 10^–6^ eV and relaxations on atoms stopped when differences in energies in consecutive structures were below 10^–5^ eV. Optimal Monkhorst–Pack **k**-points grid meshes of 7 × 7 × 7 and 7 × 7 × 1 dimensions were used for bulk and surfaces, respectively, while for isolated atoms, **Γ**-point calculations were carried out in a broken symmetry cell of 9 × 10 × 11 Å dimensions to ensure a correct orbital occupancy.

The electronic structure calculations were non-spin polarized, with the exception of ferromagnetic Fe, Ni, and Co bulk systems, their surfaces, and of any isolated TM atom. Optimizations were carried out using the tetrahedron smearing method of Blöchl *et al.*(21) with an energy width of 0.2 eV to speed up convergence for bulk and surface systems, and a Gaussian smearing of 0.001 eV width for isolated TM atoms. Final total energies were extrapolated to zero smearing. Surfaces were represented with six-layer slab models, with no fixed atoms, constructed from the bulk as-optimized structures, and adding 10 Å of vacuum to avoid interaction between repeated slab, as done in earlier works.^5,8^

As per the bulk and surface properties, one can refer to the literature for specific details on how they are gained.^4−6,8^ We briefly outline the cohesive energy, *E*_coh_, given per atom, calculated as follows:
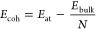
1where *E*_at_ is the energy of an atom isolated in the vacuum, and *E*_bulk_ the energy of a bulk TM containing *N* atoms. The bulk modulus, *B*_0_, was obtained by artificially enlarging/contracting the optimized bulk by ±0.05 and ±0.10 Å variations of the lattice constants, and it is defined as follows:
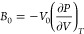
2where *V*_0_ is the unit cell volume at the ground state, and the rest of the equation is the bulk pressure change with respect to volume at constant temperature, *T*. The surface energy, γ, is defined as the energetic cost of separating the bulk by a plane and is calculated as follows:
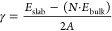
3where *E*_slab_ is the slab total energy composed of *N* atoms, *E*_bulk_ is the total energy of an atom in bulk environment, and *A* is the surface area of each one of the two equivalent exposed surfaces within a slab model. The work function, ϕ, is the minimum needed energy to move an electron from the Fermi energy, *E*_F_, to the vacuum energy level, *V*.
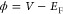
4

In order to acquire *V*, the electron electrostatic potential energy was averaged for each surface along the normal to the surface direction until a constant value was found in the vacuum region. *E*_F_ was obtained from the total density of states (DOS), sampled by *ca.* 10,000 points between the *d*-band initial and final energies.^22^ Finally, the surface relaxation, Δ_*ij*_, is obtained as a percentage relating the layer contraction/expansion at the surface
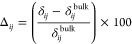
5where δ_*ij*_ is the interlayer distance of two consecutive layers in the surface and δ_*ij*_^bulk^ is the interlayer distance of two consecutive layers within the bulk. The *i* and *j* indices refer to the surface layer, *e.g.*, *i* = 1, and first subsurface layer, *e.g.*, *j* = 2.

## 3 Results and Discussion

### 3.1 Bulk Properties

Let us address first the bulk properties for the 27 TMs having either *bcc* —V, Nb, Ta, Cr, Mo, W, and Fe—, *hcp* —Sc, Y, Ti, Zr, Hf, Tc, Re, Ru, Os, Co, Zn, and Cd—, or *fcc* —Rh, Ir, Ni, Pd, Pt, Cu, Ag, and Au—crystallographic structures. Such TM bulks were fully optimized using six different *xc* functionals, HL, PZ, AM05, revPBE, SCAN, and BEEF. For each TM, three different properties were derived: the shortest interatomic distance, δ; the cohesive energy, *E*_coh_; and the bulk modulus, *B*_0_. The obtained values are listed in Tables S1–S3 of the Supporting Information and compared with experimental data available in the literature,^4,8^ duly corrected by zero point energy (ZPE) and temperature contributions. This analysis helps in evaluating the suitability and accuracy of such *xc* functionals in describing these TM bulks. For a better statistical analysis, we analyzed average deviations using the mean absolute percentage error (MAPE), listed in Table 1.

**Table 1:** Calculated MAPE Errors for the Computed Bulk Properties for Each of the Evaluated *xc* Functionals^a^

	HL	PZ	AM05	revPBE	SCAN	BEEF
δ	2.22	2.28	1.78	2.63	2.15	4.49
*E*_coh_	25.91	23.83	10.49	14.22	10.85	29.61
*B*_0_	23.68	24.11	15.21	10.03	15.72	26.10

a All values are expressed in %.

**Figure 1:**
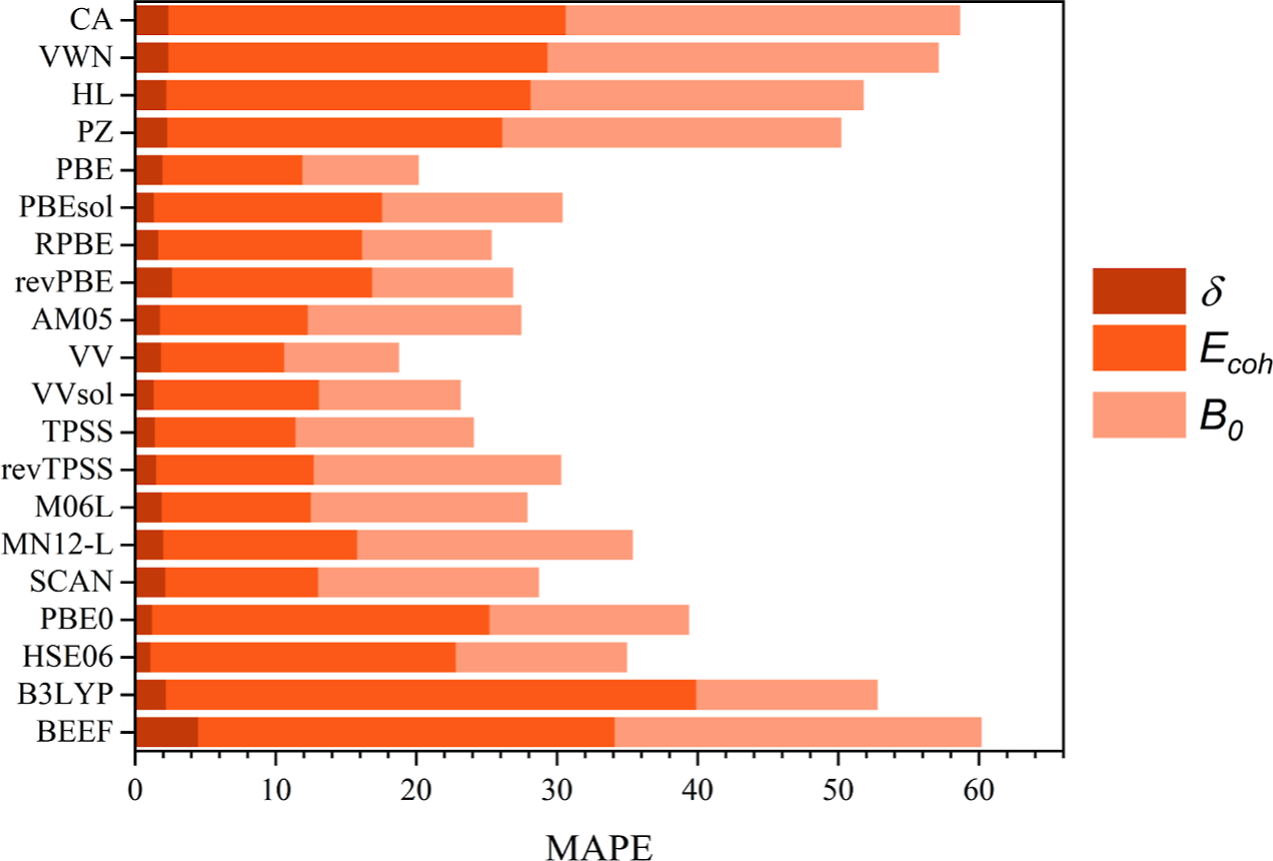
MAPE error of bulk properties for all compared functionals, including those evaluated in this study and those found in refs (4)–^6^ and (8). All the MAPE values are in %.

A close inspection of the MAPE errors in Table 1 reveals that, for the shortest interatomic distance, δ, all the inspected *xc* functionals deliver quite accurate results, with errors in the range of 1.5–2.5%. Such results are actually in line with previous studies analyzing a number of LDA, GGA, meta-GGA, and hybrid *xc* functionals,^4,6,8^ see Figure 1, in which MAPEs were in the range of 1.1–2.2%. Still, one has to mention BEEF *xc* functional as an exception, with a significantly larger MAPE of 4.49%. Notice that BEEF is ML adjusted to meet different properties, not only δ, and one could assume that the accuracy of shortest interatomic distances may be sacrificed at the expense of better describing other properties.^10^

This can be further analyzed, *e.g.*, having a close inspection to *E*_coh_ and *B*_0_ values, see Table 1 and Figure 1. As far as *E*_coh_ is concerned, there are already differences according to the Jacob’s Ladder rungs. For instance, the LDA *xc* tend to overestimate the binding strengths, and so, *E*_coh_ values tend to be larger than they should be.^6^ This trend was already observed for VWN and CA parametrizations, but consistently reproduced here by HL and PZ ones, with *E*_coh_ MAPE values of 25.91 and 23.83%, respectively. On the other side, hybrid *xc* tend to unduly destabilize the metallic, delocalized bonding, which translates in an underestimation of the *E*_coh_ values.^6^ Finally, GGA and meta-GGA perform better for *E*_coh_ values, with MAPE values in between 8.6 and 13.7%.^4,6,8^ The present GGA revPBE and AM05 results, with MAPEs of 14.22 and 10.49%, are actually in line with the previous statement, and so is SCAN meta-GGA *xc* functional, with a MAPE of 10.85%, as visually visible in Figure 1. The great outlier here is, again, BEEF, with a MAPE of 29.61%, and so, with a performance comparable to LDA or other hybrid *xc* functionals. Thus, with the analysis so far, BEEF is not well placed in describing TM bulk systems.

A similar picture can be withdrawn with bulk moduli, see Table 1 and Figure 1. Here, LDA functionals such as VWN and CA were known to deliver quite high MAPE values, above 20%^4,6^ and the same happened with HL and PZ *xc*, with MAPE values of 23.68 and 24.11%, see Table 1. This degree of accuracy is substantially increased when using GGA and meta-GGA functionals as observed in previous works.^4,8^ In general, meta-GGA *xc* functionals provided MAPEs better than LDAs, but still larger than GGAs. Take for instance SCAN *xc*, with a MAPE of 15.72%, larger than the values 10.03 and 15.21% for revPBE and AM05, respectively, and larger than previous reports on GGAs, with MAPE values going from 8.16 (VV) up to 12.84% (PBEsol).^8,23^ Finally, similar to that found for *E*_coh_, the BEEF performance is comparable to LDA *xc*, with a MAPE of 26.10% for *B*_0_ values.

Thus, after inspecting δ, *E*_coh_, and *B*_0_ properties, the overall picture walks the line of previous analyses, implying that both LDA and hybrid *xc* are not advised in the description of TM bulks, see Figure 1.^24^ The origin of the distinct *xc* accuracy behavior has, apparently, its origin in the bonding over- or underestimation. For instance, LDA *xc* functionals are well-known for their overestimation of bonding interactions. For TMs, this leads to shorter interatomic distances, and, consequently, stronger cohesive energies. In turn, the stronger interactions and deeper bond energy wells translate into more rigid materials, and so, larger bulk moduli, in consonance with the present results. On the other side of the *xc* spectrum, hybrid functionals present an underestimation of the TM bonds due to the unduly and unnatural localization of the electron density on the otherwise delocalized electronic structure of metals, leading to opposite trends as found for *xc* LDA functionals. Thus, only GGA and meta-GGA deliver more reasonable descriptions. For such rungs, VV is still unbeaten, closely followed by PBE,^8^ while the revPBE and revised PBE (RPBE) by Hammer, Hansen, and Nørskov *xc* functional resemblance is remarkable,^18,25^ and SCAN performance is similar to other meta-GGA, such as Minnesota M06-L.^26^ Here, it is worth pointing out that BEEF presents poor accuracy, comparable to LDA *xc* functionals, see Figure 1. These results already shed light on the discussion whether an improved DFT *xc* functional should (i) follow the path of accomplishing the theory constrains or be adjusted to experimental data, and (ii) whether one should focus on targeting a given property, or, at variance, diversify the adjustment to different properties, while broadening the description to different chemical environments, *e.g.*, different crystallographic phases.

**Figure 2:**
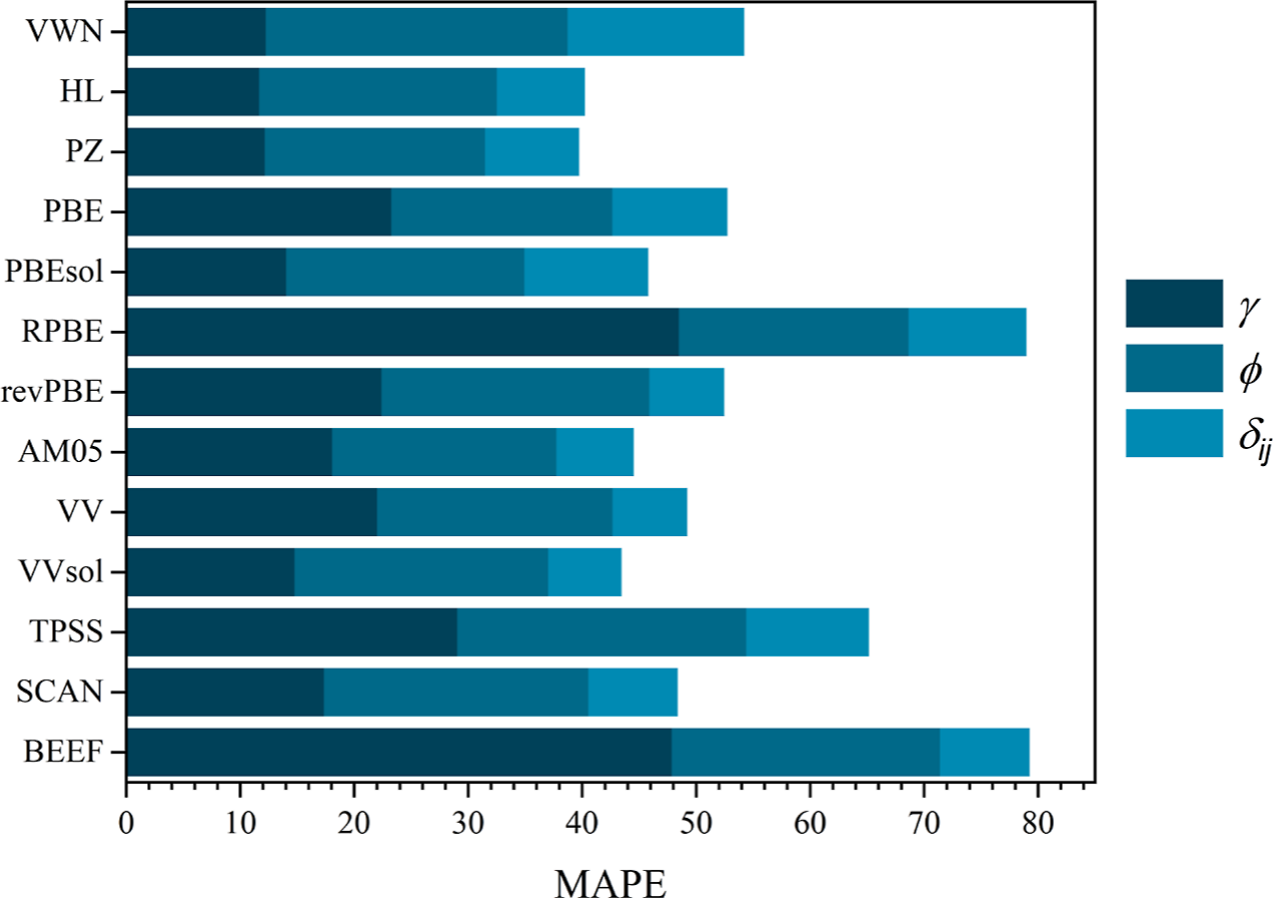
MAPE error of surface properties for all compared functionals, including those evaluated in this study and those found in refs (4), (5), and (8). All the MAPE values are in %.

Concerning the first question, it is also worth mentioning the benefits of imposing certain theory thresholds; for example, VVsol was developed by simply recovering the local spin density (LSD) response lost in PBE adapted for solids (PBEsol),^27^ leading to a better *xc* describing TM bulks.^8^ In this line, SCAN, accomplishing for 17 theoretical constrains, is, indeed, performing quite well, especially when one compares to ML parametrization as gained in BEEF. Actually, the parametrization of BEEF could be a valid and reasonable option. However, it could have been carried out aiming at simultaneously reproducing many and too different features of systems of quite different chemical nature, including covalent, ionic, and metallic systems, while underrepresenting certain solid systems, *e.g.*, only few late TMs. An adjustment based on larger and diverse properties on TM bulks family, as done on VV *xc* functional, delivers one of the best GGA *xc* functional so far in describing such systems, thanks to the experimental dataset employed in the adjustment.^8^ Still, though, the above discussion is carried out focusing on bulk properties, and nothing guarantees that a good accuracy met in bulk environments is reproduced on surface properties, a matter analyzed in the following section.

### 3.2 Surface Properties

To have a more complete view and assessment of the *xc* performance, we expanded the analysis considering three different surface properties; namely, the surface energy, γ; the work function, ϕ; and the interlayer relaxation distances, δ_*ij*_. The computed values are tabulated in Tables S4–S7 of the Supporting Information, for the 27 TMs most stable surfaces, i.e., with the lowest Miller indices,^12^ being the (001), (011), and (111) surfaces for *fcc* and *bcc* TMs, and (0001), (101̅0), and (112̅0) for *hcp* TMs. As done in earlier assessments, since surface energies and work functions experimental data arise from polycrystalline data, an average γ and ϕ are used when comparing to experimental data. Hence, the contribution to such observable of each surface has been weighted according to their expected area. This, in turn, has been derived from Wulff shapes minimizing the overall nanoparticle surface energy,^5,8^ and listed in Table S8 of the Supporting Information—more information about Wulff shapes and how they were gained can be found in the literature.^5^ Still, a comparison with data from most stable surface normally shows similar results, since such surfaces are the most expressed ones.

The MAPE summary from these three surface properties is shown in Table 2, and visually plot in Figure 2. As far as surface energies are concerned, it is worth highlighting that LDA HL and PZ *xc* functionals, with MAPEs of 11.67 and 12.15%, respectively, perform almost exactly as VWN does, with a MAPE of 12.24%. As shown in previous studies,^5^ LDA *xc* functionals are more accurate than GGA ones, with the notable exception of PBEsol and VVsol, whose MAPEs are 14.02 and 14.76%, respectively. The non-recommended RPBE leads to computing surface energies with absolute percentage mean errors of 48.49%.^5^ SCAN performance is quite acceptable, with a MAPE of 17.34%, while BEEF is not recommended, as its MAPE is 47.86%. The origin of the BEEF poor performance seems to be linked to the similarly poor performance of RPBE and hybrid functionals. As observed previously,^4^ BEEF underestimates the metal bonding in TM bulk materials, as derived from *E*_coh_ values, being more than 1 eV smaller than they should be in average, see Table S2 in the Supporting Information. This, for instance, explains the larger δ values, in average larger than 0.07 Å, and the smaller γ values, origin of the large MAPE errors. This is intimately linked to the broken-bond model when estimating γ values,^12^ in the sense that, the larger the *E*_coh_, the larger the γ values, and *vice versa*.

**Table 2:** Calculated MAPE Errors for the Computed Surface Properties for Each of the Evaluated *xc* Functionals^a^

	HL	PZ	AM05	revPBE	SCAN	BEEF
γ	11.67	12.15	18.06	22.39	17.34	47.86
ϕ	20.84	19.34	19.68	23.03	23.21	23.53
δ_***ij***_	7.72	8.25	6.79	6.55	7.82	7.87

a All values are expressed in %.

At variance, when it comes to work functions, ϕ, the explored *xc* perform similarly, even BEEF. Here, MAPE ranges from 19.34% (PZ) to 23.53% (BEEF), with no clear trends or differences between the different *xc* philosophies and DFT *xc* rungs. Actually, such values are in line with previous assessed *xc* functionals, going from 19.39% (PBE) to 26.49% (VWN).^5^ In this sense, since ϕ is defined by the Fermi energy with respect the vacuum energy level, all functionals seem to be similarly good at such estimates, although from the ones assessed here, LDA AM05 *xc* perform slightly better than the rest, with a MAPE of 19.68%. Finally, a similar picture can be drawn from δ_*ij*_ values, with similar MAPEs ranging from 6.55% (revPBE) to 8.25% (PZ), also in line with previous estimates of 6.43% (VVsol) to 13.76% for Tao–Perdew–Staroverov–Scuseria (TPSS)^28^ meta-GGA *xc* functional.^5,8^ In conclusion, the present *xc* functionals performance is good in comparison, even if LDA *xc* are known to underestimate distances because of the bond overestimation.

When having an overall view of the *xc* performance on the three explored surface properties, see Figure 2, one realizes that the LDA HL and PZ functionals actually perform slightly better than VWN, mostly due to a slight accumulative improvement in the description of ϕ and δ_*ij*_, and being the best in describing surface properties, yet not bulk properties, *vide supra*. As for GGA functionals revPBE and AM05, the overall performance is similar to other GGA types with the exception of RPBE as a reference, see Figure 2. In particular, revPBE performance is similar to that of PBE, while AM05 is almost competitive to VVsol, the latter still being the best in describing surface properties so far.^8^ SCAN *xc* is a clear improvement over other meta-GGA functionals, especially with a clear improvement in the description of surface energies, exemplified with respect to TPSS, see Figure 2. Finally, BEEF ML-based *xc* is not advised for TM surface properties, again mostly due to its inaccuracy in estimating γ values.

In a similar fashion to TM bulk properties, surface properties can also be connected to the *xc* over- or underestimation of the interatomic bonds. For instance, LDA functionals, which overestimate them, have stronger cohesive energies, which are directly connected to the surface energies, *i.e.*, the stronger the *E*_coh_, the larger the γ values.^12^ Likewise, the δ_*ij*_ may be underestimated with overbonding *xc* such as LDA. Finally, the stronger interaction may lead to more overlap between atomic orbitals, and so to broader valence bands, affecting the location of the Fermi level, and, in consequence, of the work function. As happened with bulk TM properties, hybrid *xc* may lead to the inverse trends compared to LDA functionals, while GGA and meta-GGA provide more balanced results. Notice that BEEF performance is somewhat similar to hybrid *xc* functionals, and the reason may be the adjustment to main group thermochemical data,^11^ biasing the performance toward such molecular systems, where hybrid functionals such as B3LYP excel over lower Jacob’s Ladder rungs, but at the expense of a poorer description of TM solid systems.^6^

### 3.3 Overall Performance

**Figure 3:**
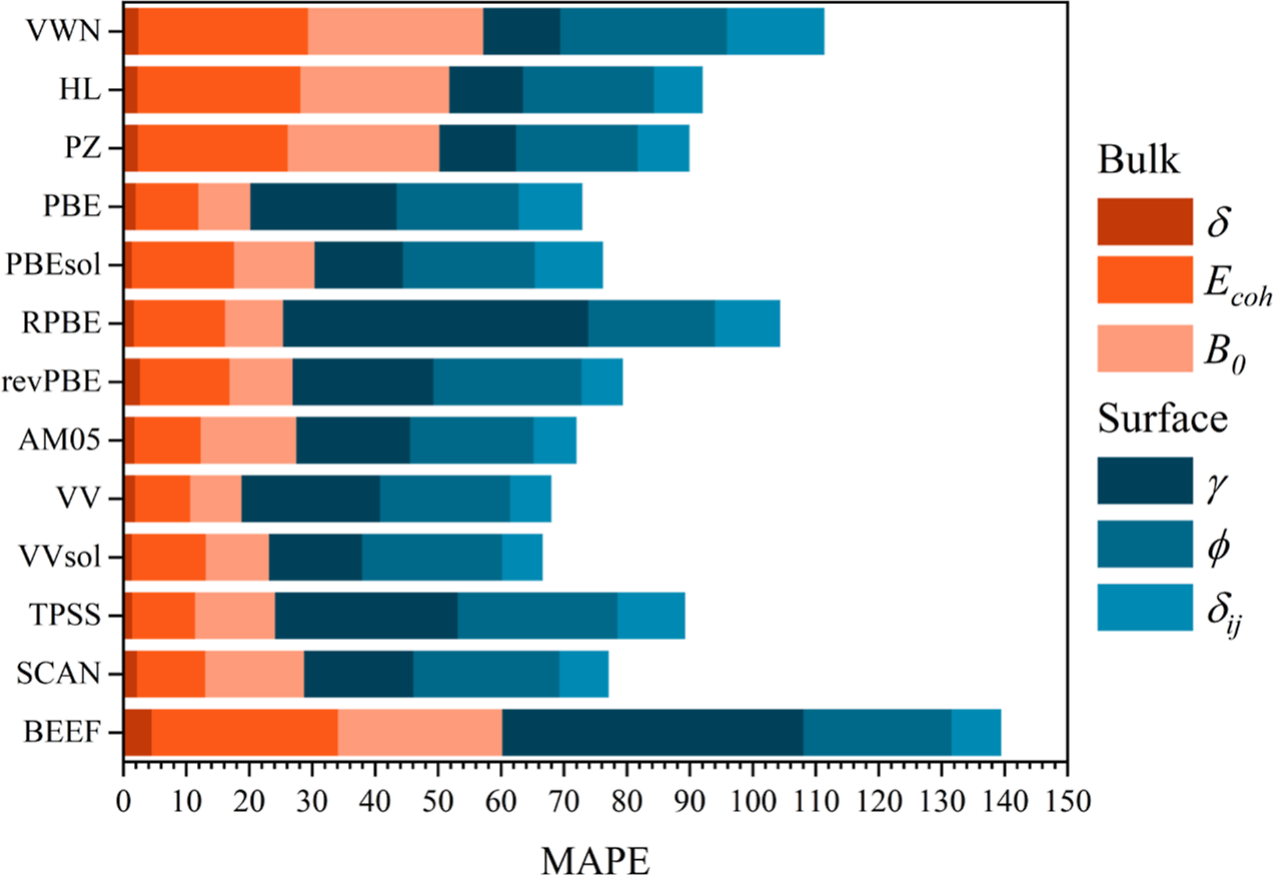
MAPE error of all evaluated bulk and surface properties for all compared functionals, including those evaluated in this study and those found in refs (4)–^6^ and (8). All the MAPE values are in %.

Having analyzed the performance of PZ, HL, revPBE, AM05, SCAN, and BEEF *xc* functionals for both a set of TM bulk and surface properties, compared to previous LDA, GGA, and meta-GGA *xc*, it is worth having a look at the overall picture, as depicted in Figure 3. As done in earlier assessments,^5,8^ we added the MAPEs of δ, *E*_coh_, *B*_0_, γ, ϕ, and δ_*ij*_, to have an overall view and compare trends against previous *xc* functionals. On one hand, VV and VVsol *xc* functionals are still the best suited in describing TM bulk and surface properties,^8^ yet closely followed by other GGA functionals, as earlier observed for PBE and PBEsol. It is worth pointing out that AM05 poses itself as a suitable option, mostly thanks to its good accuracy for surface properties. The revPBE *xc* functional performance is also good, yet slightly poorer than PBE, PBEsol, and AM05, mostly affected by the somewhat larger MAPE for workfunction values.

The accuracy of SCAN *xc* functional is sensibly better than other meta-GGA *xc* functionals, here exemplified on TPSS, see Figure 3. The SCAN theoretical adjustment is translated into significantly better description of surface properties compared to TPSS, particularly on surface energies, γ, even though the bulk properties description is somewhat poorer. Finally, BEEF ML-based *xc* functional is quite non-advised. The only properties where it has a reasonable performance are bulk shortest interatomic distance, δ, and interlayer distances, δ_*ij*_, but with accuracies on *E*_coh_ and *B*_0_ in the order of LDA *xc* functionals, adjoined with inaccuracies on γ and ϕ similar to RPBE. Clearly, even though the ML adjustment is by itself a promising, powerful tool, the resulting performance on TM material family is quite limited, asking for a much better adjustment improvement on this field.

**Figure 4:**
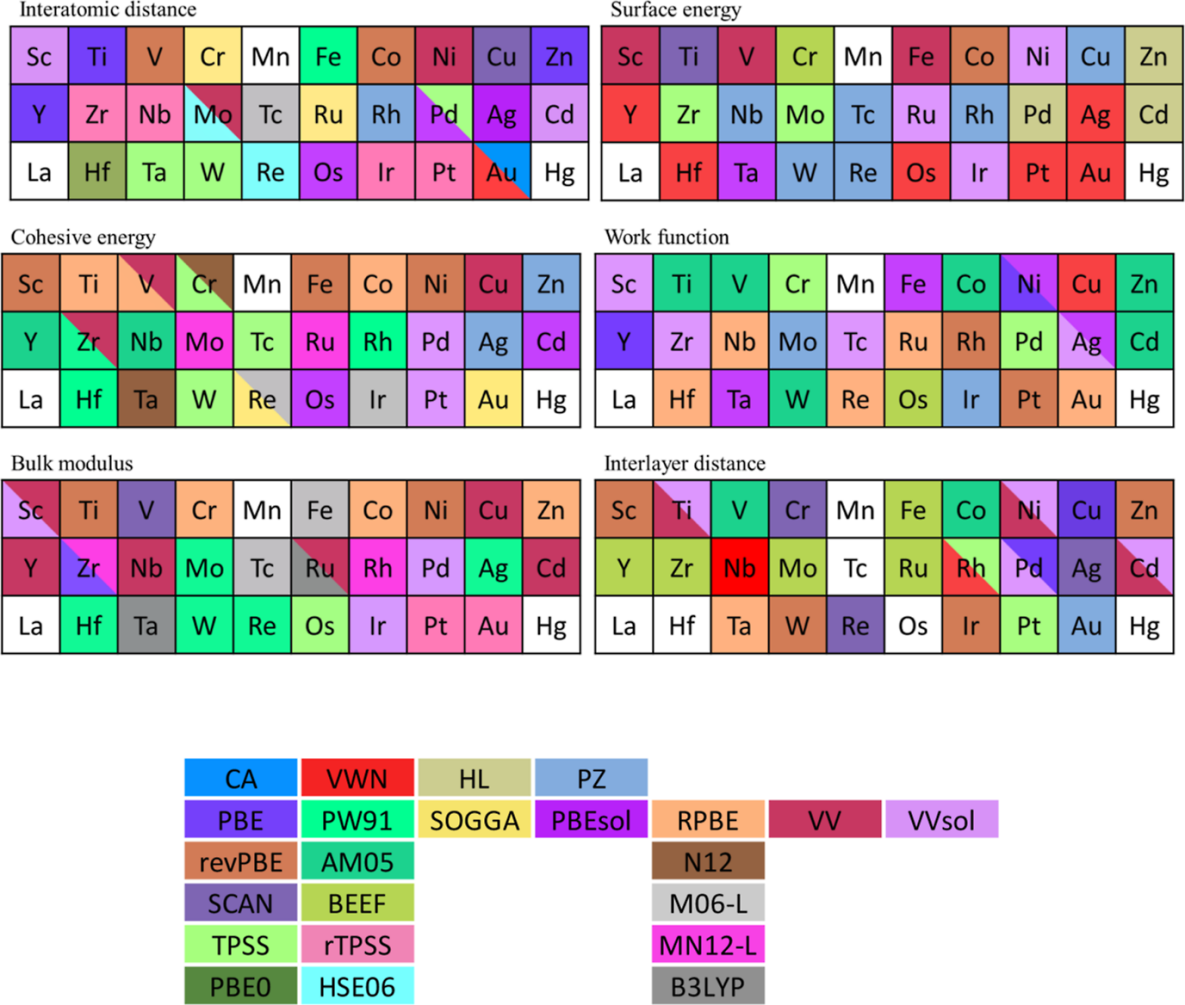
Color-coded list of *xc* functionals that yield the TM properties closest results to experimental data, extrapolated to 0 K and corrected for zero-point energies. The *xc* with similar performances, within a MAPE difference of ±0.05%, are shown as tied. Data beyond currently calculated HL, PZ, AM05, revPBE, SCAN, and BEEF is taken from refs (4)–^6^ and (8).

Last but not least, having analyzed the previous *xc* functionals, one has to stress out that none of the *xc* functionals under study is a panacea for the description of TM systems. Figure 4 shows, color coded, which explored *xc* is best at describing each property on each TM, with those functionals with similar accuracy, *i.e.*, with MAPE difference of at most ±0.05%, sharing the status. As one can see, there is absolutely no clear distribution pattern, neither through *d* series nor groups, crystallographic structures, or properties. It is as if each TM and property is a case study by itself, with its own casuistry, which puts the light focus on the drawbacks of DFT in describing different systems. Even though certain particular functionals may be most adequate in the description of TMs and certain properties in mean terms, one has to keep in mind exactly that such a claim is done in averaged terms, and underscoring, as a corollary, that there is still room for improvement in the development of DFT *xc* functions, and, even that perhaps there is no universal description, and one should narrow the *xc* development endeavors on certain systems families and/or properties.

## 4 Conclusions

Here, we explored different *xc* functionals performance in describing a list of TM bulk properties, in particular the shortest interatomic distance, δ, cohesive energy, *E*_coh_, and bulk modulus, *B*_0_, and three TM surface properties, the surface energy, γ, work function, ϕ, and interlayer relaxation distances, δ_*ij*_, for the three most stable surfaces of each TM, thus exploring 27 TMs bulks with *hcp*, *bcc*, or *fcc* crystalline structures, and 81 TM surfaces with Miller indices order maximum of one. The studied *xc* functionals were the meta-GGA SCAN and ML-built BEEF, as well as the two LDA HL and PZ *xc* functionals, and two GGA functionals, the AM05 and the revPBE.

The accuracy evaluation with respect zero kelvin extrapolated experimental data, regarding zero point energy corrections, and averaged value for nanoparticle systems following Wulff construction surface weights, reveal similarities in accuracy for LDA HL and PZ to previous functionals of the same rung, such as the VWN, with improved description of surface properties. Aside, the revPBE functional appears to work better than RPBE, yet the performance of AM05 is highlighted in comparison, in particular due to a better performance for surface properties, yet not surpassing the accuracy achieved for broadly used PBE or PBEsol functionals, or the better adjustments of VV or VVsol functionals. Finally, SCAN *xc* functional performance, accomplishing all 17 requisites an *xc* must have, is remarkable good, while, on the other hand, the ML-based BEEF functional shows deficiencies in describing bulk and surface properties, except for interatomic and interlayer distances, requiring an improvement diversification and increase in the number of the employed experimental data in the adjustment, although the absence of a constantly better functional in describing the TM material family asks as well as on focusing the *xc* development on an adjustment targeting certain properties or similar material compounds.

## Supporting Information Available

Computed shortest interatomic distance, δ, for all evaluated exchange-correlation (*xc*) functionals and transition metals; computed cohesive energy, *E*_coh_, for all evaluated *xc* functionals, in eV/atom; computed bulk modulus, *B*_0_, for all evaluated *xc* functionals; computed surface energies, γ, for all evaluated *xc* functionals; computed work function, ϕ, for all the evaluated *xc* functionals; computed interlayer relaxation distance, Δ_ij_, for all evaluated *xc* functionals; computed interlayer relaxation distance, δij, for all evaluated *xc* functionals; and computed percentage of exposed surfaces for all the evaluated *xc* functionals (PDF)
