# Supplementary material for: Quantifying the Accuracy of Density Functionals on Transition Metal Bulk and Surface Properties
Source: J Chem Theory Comput. 2023 Nov 9;19(22):8285–92. doi: 10.1021/acs.jctc.3c00612 (PMC10688177; doi:10.1021/acs.jctc.3c00612)
Supplement: Supplementary file 1 — ct3c00612_si_001.pdf [file ct3c00612_si_001.pdf]

Quantifying the Accuracy of Density Functionals on Transition Metal Bulk and Surface Properties

David Vázquez-Parga, Andrea Fernández-Martínez, Francesc Viñes*

Departament de Ciència de Materials i Química Física & Institut de Química Teòrica i Computacional (IQTCUB), Universitat de Barcelona, c/Martí i Franquès 1, 08028 Barcelona, Spain

*Corresponding author: francesc.vines@ub.edu

Table S1. Computed shortest interatomic distance, δ, for all evaluated exchange-correlation (*xc*) functionals and Transition Metals (TM). All values are given in Å. The experimental (Exp.) values are given, duly corrected by Zero Point Energy (ZPE) and finite temperature contributions.

TM	HL	PZ	AM05	revPBE	SCAN	BEEF	Exp.[a]
**Sc**	3.12	3.12	3.18	3.28	3.26	2.95	3.24
**Ti**	2.82	2.82	2.85	2.90	2.87	2.92	2.89
**V**	2.53	2.53	2.56	2.60	2.58	2.62	2.61
**Cr**	2.42	2.42	2.44	2.48	2.45	2.50	2.49
**Fe**	2.39	2.39	2.41	2.47	2.38	2.52	2.45
**Co**	2.41	2.40	2.43	2.49	2.40	2.52	2.49
**Ni**	2.42	2.42	2.45	2.51	2.42	2.55	2.48
**Cu**	2.49	2.49	2.52	2.59	2.55	2.64	2.54
**Zn**	2.87	2.87	2.90	2.97	2.91	3.07	2.65
**Y**	3.44	3.44	3.50	3.61	3.59	3.44	3.55
**Zr**	3.15	3.14	3.17	3.22	3.19	3.15	3.17
**Nb**	2.83	2.83	2.84	2.89	2.86	2.91	2.85
**Mo**	2.70	2.71	2.72	2.76	2.73	2.78	2.72
**Tc**	2.69	2.69	2.70	2.74	2.70	2.77	2.71
**Ru**	2.62	2.62	2.62	2.67	2.62	2.70	2.64
**Rh**	2.66	2.66	2.68	2.73	2.67	2.78	2.53
**Pd**	2.73	2.73	2.74	2.81	2.81	2.88	2.75
**Ag**	2.89	2.84	2.88	2.97	2.89	3.07	2.88
**Cd**	2.78	2.79	2.81	2.93	2.88	3.08	2.96
**Hf**	3.06	3.06	3.10	3.16	3.06	3.18	3.13
**Ta**	2.82	2.82	2.84	2.89	2.83	2.91	2.86
**W**	2.73	2.72	2.74	2.77	2.73	2.79	2.74
**Re**	2.72	2.72	2.73	2.76	2.73	2.78	2.56
**Os**	2.66	2.66	2.67	2.70	2.65	2.72	2.67
**Ir**	2.70	2.70	2.71	2.75	2.70	2.78	2.71
**Pt**	2.76	2.76	2.77	2.82	2.73	2.88	2.77
**Au**	2.89	2.84	2.88	2.97	2.89	3.07	2.87

[a] Ref. 1.

Table S2. Computed cohesive energy, E_{coh}, for all evaluated *xc* functionals, in eV/atom. The experimental (Exp.) values are given, duly corrected by Zero Point Energy (ZPE) and finite temperature contributions.

TM	HL	PZ	AM05	revPBE	SCAN	BEEF	Exp.[a]
**Sc**	4.85	4.88	4.37	3.79	4.34	3.16	3.93
**Ti**	6.69	6.5	5.92	5.05	5.71	4.25	4.88
**V**	7.25	7.42	6.42	4.87	5.67	4.28	5.34
**Cr**	6.53	5.65	4.42	3.49	3.99	2.81	4.15
**Fe**	6.52	6.46	5.47	4.34	5.82	3.49	4.32
**Co**	6.53	6.50	5.67	4.72	5.68	3.93	4.47
**Ni**	5.95	6.02	5.19	4.28	4.89	3.56	4.48
**Cu**	4.47	4.09	3.76	3.07	3.85	2.34	3.51
**Zn**	4.24	3.40	3.33	2.49	3.32	1.68	1.38
**Y**	4.85	4.89	4.39	3.80	4.18	4.89	4.42
**Zr**	7.30	7.38	6.69	5.82	6.16	7.38	6.32
**Nb**	8.05	8.52	7.47	6.42	6.96	5.64	7.47
**Mo**	7.55	8.13	6.80	5.63	5.70	7.86	6.84
**Tc**	8.50	8.84	7.61	6.20	6.97	5.10	7.17
**Ru**	8.54	8.77	7.52	6.03	7.76	4.82	6.80
**Rh**	7.54	7.54	6.39	5.30	6.46	4.20	5.76
**Pd**	5.03	5.03	4.10	3.19	4.16	2.22	3.93
**Ag**	3.56	2.96	2.73	2.05	2.72	2.75	2.96
**Cd**	1.91	1.37	1.35	0.81	1.32	0.14	1.18
**Hf**	7.48	7.56	6.88	5.98	6.65	5.08	6.44
**Ta**	9.56	9.65	8.86	7.74	8.79	6.68	8.11
**W**	10.39	10.61	8.78	8.40	9.65	7.19	8.83
**Re**	9.43	9.76	8.52	8.75	8.32	5.88	8.06
**Os**	10.12	10.25	9.23	7.69	9.17	6.26	8.22
**Ir**	9.18	9.27	8.12	6.64	8.08	5.31	6.96
**Pt**	6.91	6.94	6.15	4.93	6.36	3.77	5.87
**Au**	3.56	2.96	2.73	2.05	2.72	2.75	3.83

[a] Ref. 1.

Table S3. Computed bulk modulus, B_0, for all evaluated *xc* functionals, given in GPa. The experimental (Exp.) values are given, duly corrected by Zero Point Energy (ZPE) and finite temperature contributions.

TM	HL	PZ	AM05	revPBE	SCAN	BEEF	Exp.[a]
**Sc**	62.8	62.0	57.4	53.0	56.6	38.7	55.6
**Ti**	128.5	128.6	119.6	108.5	115.4	105.0	108.3
**V**	215.3	216.2	202.9	180.8	174.1	169.9	158.9
**Cr**	310.6	311.4	291.3	257.6	288.2	241.8	174.5
**Fe**	270.3	271.7	238.4	209.4	246.6	125.0	169.8
**Co**	278.3	242.1	247.8	205.5	218.8	177.7	193.0
**Ni**	258.1	263.4	233.3	182.5	256.5	153.3	185.5
**Cu**	114.3	186.0	165.3	130.2	147.4	103.9	140.3
**Zn**	194.4	196.1	171.6	125.7	153.3	78.3	69.7
**Y**	44.1	44.1	41.5	39.4	41.8	57.8	41.7
**Zr**	103.9	104.0	98.3	90.7	96.8	125.0	95.9
**Nb**	196.1	196.3	188.1	170.6	193.8	106.1	172.0
**Mo**	300.7	302.4	290.7	259.5	297.6	236.1	264.7
**Tc**	351.9	352.5	349.6	297.5	334.7	262.4	303.1
**Ru**	374.3	374.9	357.3	303.8	375.9	254.1	317.7
**Rh**	324.2	323.6	302.0	244.5	307.6	194.3	288.7
**Pd**	229.4	227.6	204.5	159.2	164.5	116.9	195.4
**Ag**	140.4	140.6	115.0	78.0	88.8	40.7	103.8
**Cd**	114.1	113.2	99.1	75.9	102.8	54.2	53.8
**Hf**	108.3	122.5	114.3	107.5	121.0	104.5	109.7
**Ta**	216.4	220.4	209.1	191.2	205.0	184.5	193.7
**W**	345.9	346.3	336.1	304.9	332.1	332.6	312.3
**Re**	420.7	421.6	415.7	371.9	374.6	342.0	368.8
**Os**	458.9	460.6	453.2	397.0	479.4	351.4	424.6
**Ir**	411.0	411.7	398.7	340.0	407.4	282.4	365.2
**Pt**	310.9	311.6	295.6	239.9	379.2	181.4	284.2
**Au**	140.4	140.6	115.0	78.0	88.8	40.7	174.8

[a] Ref. 1.

Table S4. Computed surface energies, γ, for all evaluated *xc* functionals, along with the experimental (Exp.) values. All values are given in J/m^2.

TM	Surface	HL	PZ	AM05	revPBE	SCAN	BEEF	Exp.[a]
	(0001)	1.49	1.48	1.42	1.19	1.61	0.97	
**Sc**	(10$\bar{1}$0)	1.41	1.41	1.33	1.19	1.42	0.96	1.28
	(11$\bar{2}$0)	1.46	1.46	1.36	1.23	1.48	0.96	
	(0001)	2.24	2.24	1.17	1.94	2.16	1.53	
**Ti**	(10$\bar{1}$0)	2.27	2.27	1.18	1.98	2.20	1.58	2.10
	(11$\bar{2}$0)	2.17	2.17	1.04	1.87	2.01	1.48	
	(001)	2.92	2.93	2.75	2.41	2.67	1.90	
**V**	(011)	2.90	2.90	2.79	2.43	2.70	1.94	2.55
	(111)	3.19	3.19	3.06	2.70	3.01	2.08	
	(001)	4.09	4.09	3.80	3.18	3.97	2.27	
**Cr**	(011)	3.65	3.65	3.52	3.09	3.51	2.41	2.30
	(111)	4.02	4.02	3.87	3.42	3.86	2.68	
	(001)	3.34	3.39	3.04	2.44	3.14	1.61	
**Fe**	(011)	3.06	3.91	4.22	2.39	2.90	1.62	2.48
	(111)	3.43	3.31	3.18	2.63	2.96	1.78	
	(0001)	3.26	3.17	2.42	2.89	2.56	1.40	
**Co**	(10$\bar{1}$0)	2.86	2.90	2.62	3.04	2.74	1.54	2.55
	(11$\bar{2}$0)	3.88	3.79	2.87	2.41	3.04	1.67	
	(001)	2.83	2.57	2.56	2.15	2.53	1.43	
**Ni**	(011)	2.95	2.75	2.68	2.23	2.64	1.48	2.45
	(111)	2.54	2.22	2.29	1.88	2.28	1.19	
	(001)	2.02	2.02	1.78	1.45	1.77	0.80	
**Cu**	(011)	2.08	2.08	1.84	1.50	1.80	0.82	1.83
	(111)	1.78	1.78	1.57	1.27	1.51	0.68	
	(0001)	0.71	0.71	1.57	0.44	0.41	0.03	

**Zn**	$(10\bar{1}0)$	0.91	0.92	1.28	0.65	0.74	0.22	0.91
	$(11\bar{2}0)$	1.38	1.37	1.19	0.95	1.19	0.39	
**Y**	(0001)	1.14	1.14	1.08	0.94	1.24	0.57	
	$(10\bar{1}0)$	1.13	1.13	1.06	0.96	1.14	0.67	1.13
	$(11\bar{2}0)$	1.17	1.17	1.10	1.01	1.20	0.61	
**Zr**	(0001)	1.81	1.81	1.73	1.57	1.76	1.05	
	$(10\bar{1}0)$	1.82	1.82	1.76	1.61	1.79	1.09	2.00
	$(11\bar{2}0)$	1.89	1.89	1.78	1.63	1.77	1.04	
**Nb**	(001)	2.76	2.76	2.62	2.33	2.52	1.85	
	(011)	2.46	2.46	2.38	2.13	2.32	1.68	2.70
	(111)	2.73	2.73	2.64	2.35	2.57	1.82	
**Mo**	(001)	3.43	3.43	3.34	2.98	3.38	2.30	
	(011)	3.04	3.04	2.95	2.63	2.95	2.04	3.00
	(111)	3.43	3.43	3.33	2.96	3.30	2.30	
**Tc**	(0001)	2.76	2.76	2.60	2.26	2.43	1.58	
	$(10\bar{1}0)$	3.12	3.12	2.95	2.61	2.81	1.91	3.15
	$(11\bar{2}0)$	3.25	3.25	3.15	2.70	2.92	1.95	
**Ru**	(0001)	3.09	3.10	2.88	2.53	2.90	1.80	
	$(10\bar{1}0)$	3.49	3.49	3.28	2.86	3.28	2.05	3.05
	$(11\bar{2}0)$	4.01	4.01	3.77	3.30	3.78	2.39	
**Rh**	(001)	3.40	3.40	3.20	2.79	3.17	1.93	
	(011)	3.43	3.44	3.23	2.78	3.16	1.86	2.70
	(111)	2.61	2.61	2.42	2.09	2.37	1.34	
**Pd**	(001)	2.19	2.10	1.80	1.52	1.73	0.82	
	(011)	2.24	2.17	1.85	1.52	1.70	0.80	2.05
	(111)	1.75	1.64	1.38	1.19	1.27	0.57	
**Ag**	(001)	1.30	1.29	1.01	0.78	1.00	0.31	
	(011)	1.34	1.34	1.05	0.81	1.03	0.32	1.25
	(111)	1.14	1.14	0.88	0.67	0.89	0.24	
	(0001)	0.54	0.54	0.38	0.28	0.34	0.02	

**Cd**	$(10\bar{1}0)$	0.66	0.66	0.52	0.39	0.44	0.01	0.69
	$(11\bar{2}0)$	0.94	0.94	0.74	0.54	0.67	0.13	
**Hf**	(0001)	1.92	1.92	1.87	1.69	1.79	1.38	
	$(10\bar{1}0)$	2.05	2.05	1.99	1.82	1.90	1.50	2.15
	$(11\bar{2}0)$	2.09	2.09	1.99	1.82	1.91	1.48	
**Ta**	(001)	2.91	2.92	2.80	2.49	2.77	2.01	
	(011)	2.70	2.70	2.63	2.36	2.57	1.91	3.15
	(111)	3.07	3.07	3.01	2.70	2.92	2.18	
**W**	(001)	4.25	4.25	4.20	3.82	4.15	3.16	
	(011)	3.46	3.46	3.42	3.09	3.35	2.51	3.68
	(111)	3.85	3.85	3.80	3.40	3.75	2.74	
**Re**	(0001)	3.09	3.09	3.00	2.63	3.03	1.88	
	$(10\bar{1}0)$	3.42	3.42	3.33	2.95	3.39	2.20	3.60
	$(11\bar{2}0)$	3.63	3.64	3.56	3.13	3.51	2.30	
**Os**	(0001)	3.36	3.37	3.49	2.86	3.26	2.06	
	$(10\bar{1}0)$	3.86	3.86	3.75	3.30	3.76	2.45	3.45
	$(11\bar{2}0)$	4.63	4.63	4.50	3.97	4.62	3.01	
**Ir**	(001)	2.94	2.94	2.67	2.30	2.61	1.49	
	(011)	2.99	2.97	2.71	2.29	2.61	1.46	3.00
	(111)	2.30	2.30	2.07	1.76	1.99	1.12	
**Pt**	(001)	2.47	2.48	2.24	1.90	2.28	1.11	
	(011)	2.55	2.55	2.30	1.94	2.31	1.08	2.48
	(111)	2.05	2.06	1.82	1.55	1.80	0.80	
**Au**	(001)	1.37	1.37	1.10	0.87	1.01	0.28	
	(011)	1.37	1.37	1.09	0.84	0.99	0.25	1.50
	(111)	1.10	1.10	0.86	0.68	0.79	0.17	

[a] Ref. 2.

Table S5. Computed work function, ϕ, for all the evaluated *xc* functionals, along with the experimental (Exp.) values. All values are given in eV.

TM	Surface	HL	PZ	AM05	revPBE	SCAN	BEEF	Exp.[a]
**Sc**	(0001)	3.92	3.85	4.68	3.21	4.18	3.21	3.50
	$(10\bar{1}0)$	4.30	4.22	4.54	2.31	4.31	2.31	
	$(11\bar{2}0)$	3.71	3.64	4.62	2.08	3.89	2.08	
**Ti**	(0001)	5.16	5.09	5.25	2.62	5.22	2.62	4.33
	$(10\bar{1}0)$	4.40	4.33	4.34	2.56	4.48	2.56	
	$(11\bar{2}0)$	3.94	3.87	3.66	2.06	3.89	2.06	
**V**	(001)	4.58	4.51	4.35	2.45	5.10	2.45	4.30
	(011)	5.61	5.54	3.77	3.33	5.74	3.33	
	(111)	4.61	4.53	4.06	2.68	4.46	2.68	
**Cr**	(001)	4.93	4.84	4.56	3.27	4.93	3.27	4.50
	(011)	5.70	5.61	5.24	3.51	5.81	3.51	
	(111)	5.22	5.15	4.30	3.05	5.07	3.05	
**Fe**	(001)	4.94	4.96	4.23	2.68	5.89	2.68	4.50
	(011)	5.93	5.82	5.48	3.51	6.22	3.51	
	(111)	5.28	5.89	4.10	3.17	5.89	3.17	
**Co**	(0001)	6.07	6.03	5.23	3.64	6.21	3.64	5.00
	$(10\bar{1}0)$	5.86	5.82	5.11	3.64	6.00	3.64	
	$(11\bar{2}0)$	5.56	5.43	4.72	3.34	5.46	3.34	
**Ni**	(001)	6.07	6.00	5.24	3.87	6.82	3.87	5.15
	(011)	5.68	5.60	4.71	3.46	5.68	3.46	
	(111)	6.31	6.23	5.32	3.96	6.37	3.96	
**Cu**	(001)	5.60	5.52	4.81	3.28	5.72	3.28	4.65
	(011)	5.63	5.54	4.83	3.51	5.41	3.51	
	(111)	5.76	5.68	5.21	3.50	5.73	3.50	
**Zn**	(0001)	5.21	5.13	4.21	2.82	5.21	2.82	4.33
	$(10\bar{1}0)$	5.59	5.52	4.48	3.41	5.47	3.41	
	$(11\bar{2}0)$	5.17	5.09	4.43	3.02	5.11	3.02	
**Y**	(0001)	3.65	3.59	5.61	6.30	4.18	6.30	3.10
	$(10\bar{1}0)$	4.01	3.94	5.29	2.82	4.42	2.82	

	$(11\bar{2}0)$	3.49	3.42	4.73	1.90	3.63	1.90	
**Zr**	(0001)	4.84	4.78	5.83	5.10	5.22	5.10	
	$(10\bar{1}0)$	4.54	4.47	5.31	2.71	4.53	2.71	4.05
	$(11\bar{2}0)$	3.82	3.75	5.20	1.99	3.70	1.99	
**Nb**	(001)	4.38	4.29	4.64	2.50	4.62	2.50	
	(011)	5.21	5.14	5.48	3.36	5.37	3.36	4.30
	(111)	4.57	4.50	4.31	2.68	4.45	2.68	
**Mo**	(001)	4.70	4.64	4.53	2.64	5.26	2.64	
	(011)	5.39	5.32	5.40	3.26	5.61	3.26	4.60
	(111)	5.07	4.98	4.63	3.11	5.01	3.11	
**Tc**	(0001)	5.56	5.49	5.25	3.32	5.43	3.32	
	$(10\bar{1}0)$	5.54	5.46	5.16	3.50	5.43	3.50	
	$(11\bar{2}0)$	5.20	5.13	4.76	3.28	4.90	3.28	
**Ru**	(0001)	5.96	5.89	5.73	3.90	6.16	3.90	
	$(10\bar{1}0)$	5.92	5.86	5.55	3.93	6.18	3.93	4.71
	$(11\bar{2}0)$	5.42	5.35	5.43	3.86	5.36	3.86	
**Rh**	(001)	6.62	6.57	6.44	4.73	7.05	4.73	
	(011)	6.00	5.92	6.27	4.12	6.07	4.12	4.98
	(111)	6.46	6.55	6.54	4.63	6.73	4.63	
**Pd**	(001)	6.41	6.34	5.88	4.26	6.57	4.26	
	(011)	6.07	5.99	5.32	4.01	5.66	4.01	5.12
	(111)	6.48	6.40	6.17	4.38	6.25	4.38	
**Ag**	(001)	5.48	5.39	4.86	3.36	5.54	3.36	
	(011)	5.44	5.36	4.72	3.37	5.17	3.37	4.26
	(111)	5.46	5.43	5.62	3.18	5.28	3.18	
**Cd**	(0001)	4.91	4.89	4.51	2.31	4.67	2.31	
	$(10\bar{1}0)$	5.32	5.24	4.48	2.57	5.04	2.57	4.22
	$(11\bar{2}0)$	4.92	4.84	4.46	0.42	4.93	0.42	
**Hf**	(0001)	4.97	4.90	6.03	3.37	5.17	3.37	
	$(10\bar{1}0)$	4.66	4.59	4.66	2.90	4.63	2.90	3.90

	$(11\bar{2}0)$	3.83	3.76	4.57	2.24	3.81	2.24	
**Ta**	(001)	4.54	4.47	5.28	2.80	5.34	2.80	
	(011)	5.39	5.32	6.36	3.55	5.43	3.55	4.25
	(111)	4.58	4.50	4.42	2.74	4.60	2.74	
**W**	(001)	5.05	4.98	4.72	3.01	5.71	3.01	
	(011)	5.62	5.55	5.32	3.75	5.80	3.75	4.55
	(111)	5.30	5.22	4.77	3.38	5.25	3.38	
**Re**	(0001)	5.78	5.71	6.37	3.85	5.98	3.85	
	$(10\bar{1}0)$	5.74	5.67	5.68	3.86	5.98	3.86	4.96
	$(11\bar{2}0)$	5.39	5.30	5.62	3.59	5.65	3.59	
**Os**	(0001)	6.24	6.08	6.26	5.24	6.47	4.24	
	$(10\bar{1}0)$	6.37	6.30	5.66	5.40	6.51	4.40	4.83
	$(11\bar{2}0)$	5.83	5.74	5.16	3.90	5.88	3.90	
**Ir**	(001)	6.23	6.16	5.84	4.21	6.48	4.21	
	(011)	6.05	4.93	5.39	3.65	4.91	3.65	5.27
	(111)	6.11	6.04	6.42	4.01	6.11	4.01	
**Pt**	(001)	6.60	6.77	6.61	5.88	7.01	4.88	
	(011)	6.47	6.40	6.10	5.66	6.95	4.66	5.65
	(111)	6.88	6.79	6.61	5.23	7.10	5.23	
**Au**	(001)	6.25	6.16	5.98	4.37	6.55	4.37	
	(011)	6.25	6.17	5.63	4.29	6.13	4.29	5.10
	(111)	6.29	6.20	6.52	4.16	6.15	4.16	

[a] Ref. 3.

Table S6. Computed interlayer relaxation distance, Δ_{ij}, for all evaluated *xc* functionals, along with the experimental (Exp.). All values are given in %.

TM	Surface		HL	PZ	AM05	revPBE	SCAN	BEEF	Exp.[a]
	(0001)	Δ_{12}	-3.04	-3.01	3.05	-6.36	-3.24	-5.30	-6.10
		Δ_{34}	0.66	0.69	0.88	-2.38	2.40	-0.06	-1.10
		Δ_{45}	0.16	0.19	-4.55	-2.92	-0.61	-0.88	—
		Δ_{12}	5.31	5.59	5.28	0.07	-4.76	1.19	—

**Sc**	$(10\bar{1}0)$	Δ_{34}	-11.02	-11.12	-9.15	-10.24	0.54	-9.47	—
		Δ_{45}	10.26	10.37	12.58	8.55	-2.13	10.74	—
	$(11\bar{2}0)$	Δ_{12}	-0.51	-0.46	0.07	-2.62	-8.13	-1.62	—
		Δ_{34}	-5.82	-5.81	-2.23	-6.28	16.20	-4.43	—
		Δ_{45}	4.14	4.22	3.01	3.25	-5.33	4.38	—
**Ti**	(0001)	Δ_{12}	-8.06	-8.07	-6.19	-7.92	-11.71	-7.12	-4.90
		Δ_{34}	2.00	2.04	1.54	1.85	-4.55	3.17	1.40
		Δ_{45}	-1.14	-1.12	-2.91	-3.40	-23.32	-2.43	—
	$(10\bar{1}0)$	Δ_{12}	15.92	16.12	5.96	1.23	-14.35	4.30	-6.00
		Δ_{34}	-26.53	-26.73	-12.84	-11.71	-5.26	-12.10	—
		Δ_{45}	33.25	33.25	17.64	12.16	-26.39	15.76	—
	$(11\bar{2}0)$	Δ_{12}	-5.56	-5.58	-4.84	-6.80	-14.16	-6.12	—
		Δ_{34}	-2.99	-3.00	-3.62	-4.76	-2.86	-3.39	—
		Δ_{45}	0.64	0.61	1.11	0.01	-19.48	1.14	—
**V**	(001)	Δ_{12}	-12.68	-12.67	-10.77	-14.72	-3.84	-13.88	-6.70
		Δ_{34}	-0.88	-0.88	-0.30	-1.58	-23.33	-0.65	—
		Δ_{45}	3.97	3.90	5.69	2.69	-10.77	4.11	—
	(011)	Δ_{12}	-4.99	-5.07	-1.63	-7.19	-2.10	-6.09	—
		Δ_{34}	0.71	0.69	0.76	-0.41	-20.68	1.04	—
		Δ_{45}	-0.81	-0.87	0.17	-2.36	-10.21	-0.49	—
	(111)	Δ_{12}	-18.15	-18.44	-13.06	-11.02	-4.50	-2.71	—
		Δ_{34}	-18.28	-18.20	-20.42	-18.56	-10.48	-16.27	—
		Δ_{45}	3.81	3.90	3.62	0.01	-0.88	-1.69	—
**Cr**	(001)	Δ_{12}	-15.52	-15.55	-5.91	-4.49	-1.49	-1.13	—
		Δ_{34}	6.17	6.19	2.72	2.68	-16.85	4.51	—
		Δ_{45}	-6.56	-6.55	-0.02	-1.98	0.71	-1.36	—
	(011)	Δ_{12}	-4.27	-4.32	-3.23	-5.28	1.44	-4.26	-2.20
		Δ_{34}	0.31	0.33	1.17	-0.09	67.74	0.82	-1.30
		Δ_{45}	0.24	0.23	0.98	-0.52	-15.77	0.35	—
		Δ_{12}	-23.23	-23.24	-19.00	-23.84	2.55	-14.78	—

	(111)	Δ_{34}	-33.12	-33.04	-31.87	-35.30	68.51	-41.69	—
		Δ_{45}	17.50	17.44	16.31	16.19	-8.62	22.41	—
**Fe**	(001)	Δ_{12}	-2.51	-6.82	-1.69	-3.58	-7.24	-5.54	-1.40
		Δ_{34}	2.00	1.63	1.99	1.56	-21.75	2.36	—
		Δ_{45}	-1.58	-3.21	-2.00	0.79	-24.77	1.23	—
	(011)	Δ_{12}	-1.30	-5.56	-0.72	-1.64	-9.45	-1.66	1.00
		Δ_{34}	0.00	0.22	0.95	-0.44	-2.13	0.22	0.50
		Δ_{45}	-0.72	0.38	-0.97	-1.77	-45.12	-0.71	—
	(111)	Δ_{12}	-16.96	-51.03	-9.25	-13.99	-3.71	-16.34	-16.90
		Δ_{34}	-49.67	-186.51	-18.19	-19.44	-21.53	-17.20	-9.80
		Δ_{45}	29.77	45.34	18.05	16.00	-7.55	11.68	—
**Co**	(0001)	Δ_{12}	-3.13	-2.98	-2.59	-4.64	-3.65	-3.11	2.10
		Δ_{34}	0.91	1.12	1.53	-0.05	-20.44	2.73	—
		Δ_{45}	-2.19	-2.37	-0.61	-3.89	1.79	-0.86	—
	$(10\bar{1}0)$	Δ_{12}	-19.58	-19.10	-18.27	-18.94	-4.12	-19.43	-6.50
		Δ_{34}	3.18	3.23	2.40	3.73	-13.89	2.17	1.00
		Δ_{45}	-3.65	-3.81	-2.61	-11.26	-2.76	-1.42	0.10
	$(11\bar{2}0)$	Δ_{12}	-10.74	-9.92	-10.86	-15.31	0.07	-13.23	—
		Δ_{34}	4.27	2.76	2.98	3.06	-8.49	3.85	—
		Δ_{45}	-4.52	-3.11	-0.01	-1.50	-0.18	0.51	—
**Ni**	(001)	Δ_{12}	-3.44	-3.03	-3.01	-5.49	-7.41	-3.96	-3.20
		Δ_{34}	1.00	1.30	1.33	-0.76	-5.04	1.22	—
		Δ_{45}	0.41	0.67	0.77	-1.30	-7.20	0.34	—
	(011)	Δ_{12}	-10.03	-9.20	-8.87	-12.56	-7.42	-11.20	-8.70
		Δ_{34}	2.42	2.64	1.68	1.02	-2.90	1.40	3.00
		Δ_{45}	0.00	0.31	0.68	-1.49	-7.84	1.26	-0.50
	(111)	Δ_{12}	-0.73	-0.54	-0.78	-2.41	-14.88	-1.26	-1.20
		Δ_{34}	0.17	0.20	0.18	-1.16	-6.44	-0.10	—
		Δ_{45}	0.30	0.32	0.36	-0.90	-12.09	0.09	—
		Δ_{12}	-2.96	-2.93	-2.30	-4.64	-7.24	-3.27	-1.10

	(001)	Δ_{34}	0.15	0.19	1.06	-1.26	-21.75	0.74	1.70
		Δ_{45}	-0.41	-0.51	0.30	-2.10	-24.77	-0.20	—
		Δ_{12}	-11.69	-11.59	-9.88	-13.67	-9.45	-12.35	-10.00
**Cu**	(011)	Δ_{34}	4.78	4.72	4.28	3.46	-2.13	5.14	—
		Δ_{45}	-2.35	-2.27	-2.08	-3.61	-45.12	-1.81	—
		Δ_{12}	-1.02	-1.00	-0.76	-2.32	-3.71	-1.18	-0.70
	(111)	Δ_{34}	-0.04	-0.02	0.43	-1.34	-21.53	-0.13	—
		Δ_{45}	-0.17	-0.16	0.12	-1.57	-7.55	-0.20	—
		Δ_{12}	-0.14	-0.18	0.11	-1.08	-3.65	2.55	-1.00
	(0001)	Δ_{34}	-2.81	-2.97	-2.68	-4.25	-20.44	4.20	—
		Δ_{45}	-3.27	-3.58	-3.43	-4.40	1.79	4.20	—
		Δ_{12}	68.64	68.50	27.15	67.68	-4.12	70.93	—
**Zn**	(10$\bar{1}$0)	Δ_{34}	37.27	37.00	-31.90	35.36	-13.89	41.86	—
		Δ_{45}	68.64	68.50	27.33	67.68	-2.76	70.93	—
		Δ_{12}	-18.46	-18.63	-15.33	-28.42	0.07	12.38	—
	(11$\bar{2}$0)	Δ_{34}	14.61	14.56	13.22	18.90	-8.49	9.84	—
		Δ_{45}	-6.72	-6.45	-4.69	-17.88	-0.18	4.97	—
		Δ_{12}	-2.78	-2.80	-1.84	-5.64	-7.41	4.69	—
	(0001)	Δ_{34}	1.31	1.25	1.09	-1.99	-5.04	8.26	—
		Δ_{45}	-0.97	-1.00	-0.23	-3.40	-7.20	6.12	—
		Δ_{12}	6.22	6.33	4.19	-3.56	-7.42	19.03	—
**Y**	(10$\bar{1}$0)	Δ_{34}	-9.94	-10.00	-9.33	-8.45	-2.90	-6.55	—
		Δ_{45}	10.18	10.21	9.52	5.37	-7.84	20.84	—
		Δ_{12}	-1.10	-1.02	-0.83	-3.14	-14.88	8.42	—
	(11$\bar{2}$0)	Δ_{34}	-5.20	-5.24	-5.75	-6.34	-6.44	3.07	—
		Δ_{45}	4.45	4.54	4.05	3.17	-12.09	9.93	—
		Δ_{12}	-6.60	-6.58	-7.20	-8.27	-16.45	1.24	1.60
	(0001)	Δ_{34}	0.40	0.38	0.26	0.72	-5.55	6.81	—
		Δ_{45}	1.56	1.68	-0.89	-1.40	-26.95	8.86	—
		Δ_{12}	-4.71	-5.02	-2.68	-6.18	-16.05	13.06	—

**Zr**	$(10\bar{1}0)$	Δ_{34}	-6.89	-6.69	-8.16	-7.86	-4.22	-7.43	—
		Δ_{45}	8.24	7.90	10.78	6.87	-35.66	30.96	—
	$(11\bar{2}0)$	Δ_{12}	-8.42	-8.42	-7.36	-9.13	-7.13	0.99	—
		Δ_{34}	-0.08	0.01	-1.05	-2.22	-10.11	7.48	—
		Δ_{45}	-1.45	-1.42	-0.72	-1.65	-19.42	7.96	—
**Nb**	(001)	Δ_{12}	-11.85	-11.80	-9.19	-13.92	-5.54	-13.58	-13.00
		Δ_{34}	-1.11	-0.99	-2.05	-1.53	-12.01	-0.66	—
		Δ_{45}	4.57	4.72	3.49	3.19	-2.93	3.93	—
	(011)	Δ_{12}	-4.20	-4.19	-7.01	-6.32	-2.42	-5.67	—
		Δ_{34}	0.45	0.43	1.58	-0.25	-9.34	0.54	—
		Δ_{45}	-1.18	-1.21	-0.54	-3.10	-0.15	-2.14	—
	(111)	Δ_{12}	-28.63	-28.69	-26.49	-27.73	-1.73	-27.11	—
		Δ_{34}	-18.42	-18.42	-18.88	-19.84	-11.62	-18.29	—
		Δ_{45}	11.62	11.64	10.23	8.89	0.28	6.44	—
**Mo**	(001)	Δ_{12}	-15.94	-15.92	-14.60	-17.04	-3.24	-17.07	-9.50
		Δ_{34}	4.08	4.06	3.90	4.31	2.40	6.00	—
		Δ_{45}	-4.33	-4.32	-4.82	-5.71	-0.61	-6.61	—
	(011)	Δ_{12}	-5.31	-5.31	-5.50	-6.31	-4.76	-6.29	-1.60
		Δ_{34}	0.23	0.22	2.31	-0.14	0.54	0.79	—
		Δ_{45}	0.12	0.10	-1.24	-0.52	-2.13	-0.07	—
	(111)	Δ_{12}	-25.96	-26.04	-24.93	-29.56	-8.13	-27.30	-18.00
		Δ_{34}	-31.48	-31.46	-30.65	-30.63	16.20	-26.87	4.00
		Δ_{45}	19.31	19.34	18.61	17.07	-5.33	14.32	—
**Tc**	(0001)	Δ_{12}	-6.85	-6.85	-5.85	-7.88	-11.71	-8.01	—
		Δ_{34}	4.42	4.40	4.30	4.47	-4.55	5.61	—
		Δ_{45}	-3.76	-3.76	-2.63	-4.69	-23.32	-4.86	—
	$(10\bar{1}0)$	Δ_{12}	-12.20	-12.16	-9.94	-12.45	-14.35	-11.11	—
		Δ_{34}	-1.73	-1.77	-2.84	-2.54	-5.26	-2.45	—
		Δ_{45}	1.72	1.78	3.57	1.10	-26.39	2.19	—
		Δ_{12}	-18.40	-18.36	-12.89	-20.11	-14.16	-19.09	—

	$(11\bar{2}0)$	Δ_{34}	3.90	3.88	4.31	3.71	-2.86	3.90	—
		Δ_{45}	1.48	1.48	2.15	1.11	-19.48	1.71	—
**Ru**	(0001)	Δ_{12}	-3.93	-3.95	-3.98	-4.64	-3.84	-4.43	-2.10
		Δ_{34}	0.59	0.61	0.60	0.12	-23.33	0.87	—
		Δ_{45}	1.26	1.26	1.21	0.68	-10.77	1.18	—
	$(10\bar{1}0)$	Δ_{12}	-22.39	-22.39	-18.34	-24.89	-2.10	-23.17	—
		Δ_{34}	1.61	1.60	0.27	1.06	-20.68	1.07	—
		Δ_{45}	-4.82	-4.82	-3.09	-5.44	-10.21	-3.43	—
	$(11\bar{2}0)$	Δ_{12}	-7.42	-7.45	-6.94	-8.33	-4.50	-7.47	—
		Δ_{34}	-1.94	-1.93	-2.26	-2.70	-10.48	-3.02	—
		Δ_{45}	2.89	2.90	3.12	2.64	-0.88	4.00	—
**Rh**	(001)	Δ_{12}	-4.35	-4.33	-4.59	-5.71	-1.49	-5.80	1.00
		Δ_{34}	2.18	2.18	1.78	1.08	-16.85	0.95	-0.70
		Δ_{45}	1.36	1.36	1.05	0.25	0.71	0.26	—
	(011)	Δ_{12}	-10.86	-10.75	-10.55	-12.57	1.44	-12.93	-6.90
		Δ_{34}	3.65	3.59	2.95	2.76	67.74	2.26	1.90
		Δ_{45}	-0.54	-0.48	-0.32	-1.64	-15.77	-0.59	—
	(111)	Δ_{12}	-0.78	-0.76	-0.84	-1.88	2.55	-1.96	-1.28
		Δ_{34}	1.66	1.67	1.25	0.44	68.51	0.22	-1.28
		Δ_{45}	1.82	1.86	1.94	1.00	-8.62	0.98	—
**Pd**	(001)	Δ_{12}	-1.53	-1.89	-0.74	-3.01	-11.71	-0.17	0.20
		Δ_{34}	-0.18	-0.44	0.06	-1.40	-4.55	0.85	-0.70
		Δ_{45}	-0.56	-0.86	0.10	-0.62	-23.32	1.67	—
	(011)	Δ_{12}	-10.15	-10.57	-9.08	-11.83	-14.35	-8.28	-5.10
		Δ_{34}	4.13	4.07	3.30	2.70	-5.26	3.52	2.90
		Δ_{45}	-0.72	-0.96	-0.51	-2.54	-26.39	0.56	—
	(111)	Δ_{12}	0.47	0.35	0.55	-0.71	-14.16	1.05	2.40
		Δ_{34}	-0.03	-0.09	0.13	-1.17	-2.86	0.60	0.70
		Δ_{45}	-0.31	-0.27	-0.51	-1.19	-19.48	1.62	0.70
		Δ_{12}	-1.81	-1.82	-1.52	-4.59	-3.84	-1.57	—

	(001)	Δ_{34}	0.14	0.16	0.38	-2.26	-23.33	0.78	—
		Δ_{45}	-0.29	-0.40	0.10	-2.75	-10.77	0.46	—
**Ag**	(011)	Δ_{12}	-9.66	-9.55	-8.80	-13.14	-2.10	-10.59	-7.80
		Δ_{34}	4.00	3.97	3.69	2.99	-20.68	5.47	—
		Δ_{45}	-2.00	-1.93	-1.97	-5.03	-10.21	-2.80	—
	(111)	Δ_{12}	-0.76	-0.77	-0.51	-2.74	-4.50	0.35	-0.50
		Δ_{34}	-0.26	-0.24	-0.13	-2.32	-10.48	-0.09	-0.40
		Δ_{45}	-0.15	-0.22	0.21	-2.27	-0.88	-0.37	—
**Cd**	(0001)	Δ_{12}	-0.71	-0.18	-0.05	-1.33	-1.49	4.66	—
		Δ_{34}	-2.07	-1.08	-0.74	-3.52	-16.85	7.35	—
		Δ_{45}	-2.50	-1.17	-0.78	-3.89	0.71	8.32	—
	(10$\bar{1}$0)	Δ_{12}	68.32	68.30	70.55	68.54	1.44	41.67	—
		Δ_{34}	36.64	36.60	41.10	37.07	67.74	8.77	—
		Δ_{45}	68.32	68.30	70.55	68.54	-15.77	29.69	—
	(11$\bar{2}$0)	Δ_{12}	-13.85	-14.29	-5.41	-9.19	2.55	50.97	—
		Δ_{34}	14.66	14.79	12.25	14.06	68.51	36.17	—
		Δ_{45}	-8.75	-9.19	-2.73	-6.06	-8.62	37.04	-6.10
**Hf**	(0001)	Δ_{12}	-6.87	-6.83	-7.01	-8.51	-7.24	-7.82	—
		Δ_{34}	2.69	2.71	2.80	2.33	-21.75	3.73	—
		Δ_{45}	-1.20	-1.18	-1.68	-3.45	-24.77	-2.72	—
	(10$\bar{1}$0)	Δ_{12}	-3.06	-2.81	-4.23	-7.37	-9.45	-3.45	—
		Δ_{34}	-8.01	-8.11	-6.57	-6.83	-2.13	-7.13	—
		Δ_{45}	10.95	11.24	7.48	4.64	-45.12	9.11	—
	(11$\bar{2}$0)	Δ_{12}	-7.78	-7.74	-7.08	-8.98	-3.71	-8.51	—
		Δ_{34}	-1.55	-1.50	-1.98	-3.07	-21.53	-1.82	—
		Δ_{45}	-0.67	-0.65	-0.15	-0.83	-7.55	0.41	—
	(001)	Δ_{12}	-13.00	-12.94	-12.67	-15.17	-3.65	-14.99	-11.00
		Δ_{34}	-0.90	-0.86	-1.33	-1.68	-20.44	-0.74	—
		Δ_{45}	3.82	3.91	4.38	3.46	1.79	4.08	—
		Δ_{12}	-4.79	-4.75	-2.66	-6.16	-4.12	-5.68	—

**Ta**	(011)	Δ_{34}	-0.01	0.00	0.20	-0.59	-13.89	0.20	—
		Δ_{45}	-0.81	-0.81	-0.58	-1.70	-2.76	-0.60	—
	(111)	Δ_{12}	-26.56	-26.50	-25.20	-27.34	0.07	-27.08	—
		Δ_{34}	-23.69	-23.68	-23.88	-25.36	-8.49	-24.38	—
		Δ_{45}	16.04	16.08	15.50	15.21	-0.18	14.67	—
**W**	(001)	Δ_{12}	-15.59	-15.54	-14.69	-16.45	-7.41	-16.68	-5.50
		Δ_{34}	2.99	2.95	3.09	3.54	-5.04	5.31	—
		Δ_{45}	-2.97	-2.93	-3.00	-4.74	-7.20	-5.82	—
	(011)	Δ_{12}	-3.98	-4.00	-3.28	-4.39	-7.42	-4.14	-3.10
		Δ_{34}	0.06	0.05	1.78	-0.16	-2.90	0.41	—
		Δ_{45}	-0.08	-0.08	-0.37	-0.53	-7.84	-0.17	—
	(111)	Δ_{12}	-37.38	-37.35	-36.38	-37.32	-14.88	-37.35	—
		Δ_{34}	-37.02	-37.01	-35.67	-37.68	-6.44	-36.07	—
		Δ_{45}	24.51	24.50	24.34	23.56	-12.09	22.63	—
**Re**	(0001)	Δ_{12}	-6.60	-6.64	-6.44	-7.21	-16.45	-7.62	-5.00
		Δ_{34}	4.01	4.02	4.32	3.84	-5.55	5.26	—
		Δ_{45}	-3.56	-3.58	-3.27	-4.05	-26.95	-4.57	—
	$(10\bar{1}0)$	Δ_{12}	-16.92	-16.86	-16.71	-19.36	-16.05	-17.52	-11.00
		Δ_{34}	-0.71	-0.73	-0.76	-0.39	-4.22	-0.65	1.50
		Δ_{45}	0.51	0.53	1.00	-1.88	-35.66	-0.49	—
	$(11\bar{2}0)$	Δ_{12}	-22.55	-22.53	-21.70	-24.11	-7.13	-24.98	—
		Δ_{34}	5.58	5.55	5.29	5.64	-10.11	6.42	—
		Δ_{45}	1.25	1.28	1.28	0.58	-19.42	1.44	—
**Os**	(0001)	Δ_{12}	-4.01	-4.02	0.63	-4.36	-5.54	-4.17	—
		Δ_{34}	-0.07	-0.06	-4.14	-0.43	-12.01	0.00	—
		Δ_{45}	1.74	1.77	4.27	1.43	-2.93	1.95	—
	$(10\bar{1}0)$	Δ_{12}	-23.55	-23.64	-20.51	-26.41	-2.42	-25.93	—
		Δ_{34}	3.43	3.41	4.65	3.42	-9.34	3.78	—
		Δ_{45}	-10.99	-10.70	-19.41	-12.58	-0.15	-11.52	—
		Δ_{12}	-8.95	-8.99	-8.59	-10.00	-1.73	-9.00	—

	$(11\bar{2}0)$	Δ_{34}	-1.02	-1.03	-1.16	-1.09	-11.62	-1.24	—
		Δ_{45}	1.27	1.28	3.04	0.61	0.28	1.79	—
**Ir**	(001)	Δ_{12}	-5.96	-5.83	-5.43	-6.13	-3.24	-4.44	-3.60
		Δ_{34}	-1.10	-1.12	-1.06	-1.54	2.40	-0.61	—
		Δ_{45}	-0.66	-0.69	-0.36	-0.83	-0.61	0.85	—
	(011)	Δ_{12}	-12.36	-12.44	-11.56	-12.79	-4.76	-10.77	-7.00
		Δ_{34}	-1.06	-1.04	-1.13	-1.21	0.54	-1.50	—
		Δ_{45}	-0.28	-0.25	0.17	-0.09	-2.13	4.13	—
	(111)	Δ_{12}	-3.05	-3.08	-2.76	-3.25	-8.13	-2.18	-2.60
		Δ_{34}	-1.42	-1.42	-1.03	-1.59	16.20	-0.58	—
		Δ_{45}	-0.36	-0.44	0.27	-0.38	-5.33	0.97	—
**Pt**	(001)	Δ_{12}	-2.99	-2.99	-2.74	-3.88	-16.45	-2.07	0.20
		Δ_{34}	-0.63	-0.62	-0.12	-1.70	-5.55	-0.68	—
		Δ_{45}	-0.19	-0.17	0.88	-1.25	-26.95	-0.46	—
	(011)	Δ_{12}	-15.14	-15.08	-14.28	-17.25	-16.05	-15.75	-18.50
		Δ_{34}	7.87	7.83	7.65	7.82	-4.22	8.32	-24.20
		Δ_{45}	-3.30	-3.31	-2.68	-4.11	-35.66	-1.41	—
	(111)	Δ_{12}	1.42	1.41	1.53	0.74	-7.13	2.37	1.40
		Δ_{34}	-0.68	-0.65	-0.52	-1.74	-10.11	-0.22	—
		Δ_{45}	-0.57	-0.57	-0.47	-1.69	-19.42	-0.35	—
**Au**	(001)	Δ_{12}	-1.49	-1.40	-1.66	-3.53	-5.54	-1.17	—
		Δ_{34}	-0.05	0.08	0.05	-1.98	-12.01	0.03	—
		Δ_{45}	-0.15	-0.04	0.06	-1.99	-2.93	0.14	—
	(011)	Δ_{12}	-14.88	-14.77	-13.62	-18.93	-2.42	-20.35	-20.10
		Δ_{34}	7.85	7.79	7.51	7.91	-9.34	11.65	-6.20
		Δ_{45}	-4.97	-4.94	-4.45	-8.43	-0.15	-8.78	—
	(111)	Δ_{12}	0.59	0.55	0.41	-0.92	-1.73	1.73	3.30
		Δ_{34}	-0.13	-0.11	0.02	-1.72	-11.62	-0.31	-0.80
		Δ_{45}	-0.28	-0.24	-0.41	-1.89	0.28	-1.01	—

[a] Ref. 4.

Table S7. Computed interlayer relaxation distance, δ_{ij}, for all evaluated *xc* functionals. All values are given in Å.

TM	Surface		HL	PZ	AM05	revPBE	SCAN	BEEF
Sc	(0001)	δ_{12}	2.42	2.42	2.63	2.51	2.58	2.54
		δ_{23}	2.51	2.51	2.58	2.61	2.65	2.68
		δ_{34}	2.50	2.50	2.44	2.59	2.62	2.66
	$(10\bar{1}0)$	δ_{12}	0.99	0.99	1.00	0.96	0.97	0.97
		δ_{23}	1.68	1.68	1.74	1.74	1.74	1.75
		δ_{34}	1.04	1.04	1.08	1.05	1.07	1.07
	$(11\bar{2}0)$	δ_{12}	1.61	1.61	1.64	1.62	1.63	1.64
		δ_{23}	1.53	1.53	1.61	1.56	1.57	1.59
		δ_{34}	1.69	1.69	1.69	1.72	1.74	1.74
Ti	(0001)	δ_{12}	2.12	2.11	2.18	2.18	2.14	2.21
		δ_{23}	2.33	2.33	2.35	2.40	2.39	2.44
		δ_{34}	2.26	2.26	2.24	2.27	2.25	2.31
	$(10\bar{1}0)$	δ_{12}	0.98	0.98	0.89	0.86	1.01	0.89
		δ_{23}	1.30	1.30	1.48	1.52	1.34	1.52

		δ_{34}	1.23	1.23	1.01	0.97	1.25	1.01
	$(11\bar{2}0)$	δ_{12}	1.35	1.35	1.38	1.38	1.39	1.39
		δ_{23}	1.39	1.39	1.40	1.41	1.40	1.43
		δ_{34}	1.44	1.44	1.46	1.48	1.46	1.50
**V**	(001)	δ_{12}	1.30	1.30	1.33	1.31	1.30	1.33
		δ_{23}	1.45	1.45	1.47	1.48	1.47	1.50
		δ_{34}	1.52	1.52	1.57	1.54	1.55	1.58
	(011)	δ_{12}	1.97	1.97	2.05	1.98	1.98	2.02
		δ_{23}	2.08	2.08	2.10	2.12	2.11	2.16
		δ_{34}	2.05	2.05	2.09	2.08	2.07	2.13
	(111)	δ_{12}	0.71	0.71	0.75	0.78	0.77	0.85
		δ_{23}	0.71	0.71	0.71	0.73	0.73	0.75
		δ_{34}	0.88	0.88	0.88	0.87	0.84	0.86
**Cr**	(001)	δ_{12}	1.21	1.21	1.33	1.37	1.23	1.43
		δ_{23}	1.49	1.49	1.45	1.47	1.50	1.51
		δ_{34}	1.31	1.31	1.41	1.40	1.33	1.42
	(011)	δ_{12}	1.90	1.89	1.93	1.92	1.94	1.95
		δ_{23}	1.98	1.98	2.01	2.02	2.01	2.06
		δ_{34}	1.98	1.98	2.01	2.01	2.00	2.05
	(111)	δ_{12}	0.65	0.65	0.68	0.67	0.68	0.72
		δ_{23}	0.61	0.61	0.62	0.61	0.61	0.59
		δ_{34}	0.98	0.98	0.97	0.99	0.99	1.07
**Fe**	(001)	δ_{12}	1.34	1.29	1.37	1.37	1.25	1.38
		δ_{23}	1.41	1.40	1.42	1.45	1.40	1.49
		δ_{34}	1.36	1.34	1.36	1.44	1.33	1.47
	(011)	δ_{12}	1.92	1.85	1.95	1.98	1.90	2.02
		δ_{23}	1.95	1.95	1.99	2.01	1.93	2.06
		δ_{34}	1.93	1.96	1.95	1.98	1.93	2.04
	(111)	δ_{12}	0.68	0.53	0.74	0.72	0.55	0.72
		δ_{23}	0.53	0.28	0.68	0.69	0.29	0.72

		δ_{34}	1.13	1.46	0.98	0.98	1.44	0.95
**Co**	(0001)	δ_{12}	1.90	1.89	1.93	1.93	1.91	1.98
		δ_{23}	1.97	1.97	2.01	2.02	1.98	2.10
		δ_{34}	1.91	1.91	1.96	1.94	1.94	2.03
	$(10\bar{1}0)$	δ_{12}	0.59	0.59	0.60	0.61	0.58	0.62
		δ_{23}	1.45	1.45	1.45	1.51	1.46	1.50
		δ_{34}	0.68	0.68	0.69	0.65	0.68	0.73
	$(11\bar{2}0)$	δ_{12}	1.10	1.11	1.11	1.09	1.10	1.13
		δ_{23}	1.27	1.25	1.27	1.30	1.25	1.33
		δ_{34}	1.16	1.18	1.23	1.24	1.21	1.28
**Ni**	(001)	δ_{12}	1.66	1.66	1.68	1.68	1.67	1.73
		δ_{23}	1.73	1.73	1.75	1.76	1.75	1.82
		δ_{34}	1.72	1.72	1.74	1.75	1.74	1.81
	(011)	δ_{12}	1.10	1.11	1.12	1.11	1.11	1.14
		δ_{23}	1.24	1.24	1.24	1.27	1.25	1.29
		δ_{34}	1.21	1.21	1.23	1.24	1.21	1.29
	(111)	δ_{12}	1.97	1.97	1.98	2.00	1.97	2.05
		δ_{23}	1.98	1.98	2.00	2.02	1.99	2.08
		δ_{34}	1.99	1.98	2.00	2.03	1.99	2.08
**Cu**	(001)	δ_{12}	1.71	1.71	1.74	1.75	1.73	1.81
		δ_{23}	1.77	1.77	1.80	1.81	1.82	1.88
		δ_{34}	1.76	1.75	1.79	1.79	1.80	1.86
	(011)	δ_{12}	1.12	1.12	1.15	1.14	1.12	1.18
		δ_{23}	1.31	1.31	1.32	1.34	1.32	1.39
		δ_{34}	1.22	1.22	1.23	1.25	1.24	1.30
	(111)	δ_{12}	2.02	2.02	2.04	2.07	2.02	2.13
		δ_{23}	2.04	2.04	2.07	2.09	2.07	2.15
		δ_{34}	2.03	2.03	2.06	2.08	2.08	2.15
	(0001)	δ_{12}	2.35	2.35	2.37	2.46	2.50	2.72
		δ_{23}	2.29	2.29	2.31	2.38	2.49	2.77

		δ_{34}	2.28	2.27	2.29	2.38	2.42	2.77
**Zn**	$(10\bar{1}0)$	δ_{12}	2.37	2.37	1.04	2.40	2.31	2.71
		δ_{23}	2.37	2.37	1.15	2.40	2.31	2.71
		δ_{34}	2.37	2.37	1.04	2.40	2.31	2.71
	$(11\bar{2}0)$	δ_{12}	1.09	1.09	1.14	1.05	1.11	1.55
		δ_{23}	1.51	1.51	1.51	1.66	1.54	1.51
		δ_{34}	1.21	1.21	1.25	1.14	1.18	1.43
**Y**	(0001)	δ_{12}	2.70	2.70	2.76	2.78	2.79	2.91
		δ_{23}	2.81	2.81	2.84	2.87	2.86	3.02
		δ_{34}	2.75	2.74	2.81	2.84	2.84	2.95
	$(10\bar{1}0)$	δ_{12}	1.09	1.09	1.09	1.02	1.05	1.26
		δ_{23}	1.85	1.85	1.91	1.95	1.94	1.91
		δ_{34}	1.14	1.13	1.15	1.12	1.15	1.29
	$(11\bar{2}0)$	δ_{12}	1.75	1.75	1.79	1.77	1.77	1.93
		δ_{23}	1.68	1.68	1.71	1.72	1.73	1.82
		δ_{34}	1.85	1.85	1.88	1.89	1.90	1.96
**Zr**	(0001)	δ_{12}	2.41	2.41	2.40	2.42	2.41	2.61
		δ_{23}	2.58	2.58	2.58	2.64	2.65	2.76
		δ_{34}	2.61	2.61	2.56	2.58	2.57	2.83
	$(10\bar{1}0)$	δ_{12}	0.87	0.86	0.90	0.88	0.88	1.04
		δ_{23}	1.70	1.70	1.70	1.74	1.75	1.69
		δ_{34}	0.99	0.98	1.03	1.01	0.99	1.31
	$(11\bar{2}0)$	δ_{12}	1.45	1.45	1.48	1.49	1.50	1.58
		δ_{23}	1.57	1.57	1.58	1.59	1.58	1.70
		δ_{34}	1.55	1.55	1.58	1.60	1.61	1.70
**Nb**	(001)	δ_{12}	1.46	1.46	1.50	1.46	1.48	1.48
		δ_{23}	1.61	1.62	1.61	1.64	1.63	1.67
		δ_{34}	1.71	1.71	1.70	1.72	1.73	1.75
	(011)	δ_{12}	2.21	2.21	2.17	2.22	2.23	2.25
		δ_{23}	2.32	2.32	2.36	2.35	2.34	2.39

		δ_{34}	2.28	2.28	2.31	2.29	2.29	2.33
	(111)	δ_{12}	0.73	0.73	0.75	0.75	0.77	0.76
		δ_{23}	0.80	0.80	0.80	0.80	0.79	0.82
		δ_{34}	1.07	1.07	1.06	1.06	1.10	1.04
**Mo**	(001)	δ_{12}	1.35	1.35	1.37	1.36	1.36	1.37
		δ_{23}	1.63	1.63	1.63	1.66	1.65	1.71
		δ_{34}	1.50	1.50	1.50	1.51	1.50	1.50
	(011)	δ_{12}	2.10	2.10	2.10	2.12	2.12	2.13
		δ_{23}	2.22	2.22	2.27	2.25	2.23	2.29
		δ_{34}	2.22	2.21	2.19	2.24	2.22	2.27
	(111)	δ_{12}	0.72	0.72	0.73	0.71	0.72	0.73
		δ_{23}	0.69	0.69	0.69	0.70	0.70	0.73
		δ_{34}	1.12	1.12	1.11	1.11	1.12	1.08
**Tc**	(0001)	δ_{12}	2.04	2.04	2.07	2.06	2.05	2.08
		δ_{23}	2.29	2.28	2.29	2.33	2.30	2.38
		δ_{34}	2.11	2.10	2.14	2.12	2.11	2.14
	$(10\bar{1}0)$	δ_{12}	0.70	0.70	0.72	0.71	0.71	0.73
		δ_{23}	1.54	1.54	1.53	1.56	1.53	1.57
		δ_{34}	0.80	0.80	0.82	0.81	0.81	0.82
	$(11\bar{2}0)$	δ_{12}	1.15	1.15	1.21	1.15	1.14	1.17
		δ_{23}	1.41	1.41	1.42	1.44	1.43	1.45
		δ_{34}	1.38	1.38	1.39	1.40	1.38	1.42
**Ru**	(0001)	δ_{12}	2.02	2.02	2.03	2.05	2.03	2.07
		δ_{23}	2.12	2.12	2.12	2.15	2.12	2.19
		δ_{34}	2.13	2.13	2.14	2.16	2.13	2.19
	$(10\bar{1}0)$	δ_{12}	0.63	0.63	0.66	0.63	0.64	0.65
		δ_{23}	1.58	1.58	1.56	1.60	1.58	1.62
		δ_{34}	0.74	0.74	0.76	0.75	0.75	0.78
	$(11\bar{2}0)$	δ_{12}	1.25	1.25	1.26	1.27	1.25	1.29
		δ_{23}	1.32	1.32	1.32	1.34	1.32	1.35

		δ_{34}	1.39	1.39	1.39	1.41	1.39	1.45
**Rh**	(001)	δ_{12}	1.81	1.80	1.81	1.83	1.81	1.86
		δ_{23}	1.93	1.93	1.93	1.95	1.93	1.98
		δ_{34}	1.91	1.91	1.91	1.93	1.91	1.97
	(011)	δ_{12}	1.20	1.20	1.21	1.21	1.21	1.23
		δ_{23}	1.38	1.38	1.38	1.40	1.38	1.42
		δ_{34}	1.32	1.33	1.33	1.34	1.33	1.38
	(111)	δ_{12}	2.16	2.16	2.17	2.19	2.16	2.22
		δ_{23}	2.21	2.21	2.21	2.24	2.21	2.27
		δ_{34}	2.22	2.22	2.23	2.25	2.22	2.29
**Pd**	(001)	δ_{12}	1.90	1.89	1.93	1.93	1.95	2.03
		δ_{23}	1.92	1.92	1.94	1.96	1.99	2.05
		δ_{34}	1.92	1.91	1.94	1.98	1.99	2.07
	(011)	δ_{12}	1.24	1.23	1.26	1.26	1.26	1.33
		δ_{23}	1.42	1.42	1.42	1.45	1.46	1.49
		δ_{34}	1.35	1.35	1.37	1.37	1.39	1.45
	(111)	δ_{12}	2.24	2.23	2.25	2.28	2.30	2.38
		δ_{23}	2.22	2.23	2.24	2.27	2.30	2.37
		δ_{34}	2.22	2.22	2.23	2.27	2.29	2.39
**Ag**	(001)	δ_{12}	1.97	1.97	2.01	2.01	2.04	2.14
		δ_{23}	2.01	2.01	2.04	2.06	2.05	2.19
		δ_{34}	2.00	2.00	2.04	2.05	2.03	2.18
	(011)	δ_{12}	1.29	1.30	1.32	1.31	1.33	1.39
		δ_{23}	1.48	1.48	1.49	1.53	1.51	1.62
		δ_{34}	1.39	1.39	1.41	1.42	1.40	1.49
	(111)	δ_{12}	2.30	2.30	2.34	2.36	2.36	2.52
		δ_{23}	2.31	2.31	2.35	2.37	2.35	2.51
		δ_{34}	2.31	2.31	2.36	2.37	2.34	2.50
	(0001)	δ_{12}	2.71	2.72	2.77	2.83	2.87	3.19
		δ_{23}	2.67	2.70	2.75	2.77	2.79	3.28

		δ_{34}	2.66	2.70	2.75	2.76	2.73	3.32
**Cd**	$(10\bar{1}0)$	δ_{12}	2.66	2.66	2.90	2.81	2.74	1.61
		δ_{23}	2.66	2.66	2.90	2.81	2.74	2.06
		δ_{34}	2.66	2.66	2.90	2.81	2.74	1.33
	$(11\bar{2}0)$	δ_{12}	1.28	1.28	1.40	1.40	1.37	3.31
		δ_{23}	1.71	1.71	1.69	1.78	1.74	2.54
		δ_{34}	1.34	1.34	1.44	1.44	1.45	2.58
**Hf**	(0001)	δ_{12}	2.31	2.31	2.34	2.35	2.30	2.38
		δ_{23}	2.54	2.54	2.58	2.61	2.56	2.67
		δ_{34}	2.44	2.44	2.46	2.47	2.43	2.50
	$(10\bar{1}0)$	δ_{12}	0.87	0.88	0.87	0.87	0.88	0.90
		δ_{23}	1.67	1.67	1.71	1.74	1.67	1.75
		δ_{34}	1.01	1.01	0.98	0.97	1.01	1.03
	$(11\bar{2}0)$	δ_{12}	1.45	1.45	1.47	1.48	1.45	1.49
		δ_{23}	1.54	1.54	1.55	1.56	1.55	1.59
		δ_{34}	1.55	1.55	1.58	1.60	1.56	1.63
**Ta**	(001)	δ_{12}	1.44	1.44	1.46	1.45	1.43	1.46
		δ_{23}	1.61	1.61	1.62	1.64	1.61	1.67
		δ_{34}	1.69	1.69	1.71	1.73	1.69	1.75
	(011)	δ_{12}	2.20	2.20	2.26	2.22	2.19	2.25
		δ_{23}	2.30	2.30	2.32	2.34	2.30	2.38
		δ_{34}	2.29	2.28	2.31	2.32	2.26	2.36
	(111)	δ_{12}	0.74	0.74	0.76	0.76	0.75	0.76
		δ_{23}	0.76	0.76	0.76	0.77	0.76	0.78
		δ_{34}	1.12	1.12	1.12	1.14	1.12	1.14
**W**	(001)	δ_{12}	1.36	1.36	1.38	1.37	1.36	1.38
		δ_{23}	1.62	1.62	1.63	1.66	1.61	1.70
		δ_{34}	1.53	1.53	1.53	1.53	1.54	1.52
	(011)	δ_{12}	2.14	2.14	2.16	2.17	2.14	2.19
		δ_{23}	2.23	2.23	2.27	2.26	2.22	2.29

		δ_{34}	2.22	2.22	2.22	2.25	2.22	2.27
	(111)	δ_{12}	0.66	0.66	0.67	0.67	0.67	0.68
		δ_{23}	0.66	0.66	0.67	0.67	0.67	0.68
		δ_{34}	1.20	1.20	1.20	1.21	1.18	1.20
**Re**	(0001)	δ_{12}	2.08	2.08	2.08	2.10	2.07	2.10
		δ_{23}	2.31	2.31	2.32	2.34	2.33	2.39
		δ_{34}	2.14	2.14	2.15	2.16	2.14	2.16
	$(10\bar{1}0)$	δ_{12}	0.68	0.68	0.68	0.67	0.65	0.69
		δ_{23}	1.57	1.57	1.57	1.60	1.59	1.60
		δ_{34}	0.80	0.80	0.80	0.79	0.80	0.80
	$(11\bar{2}0)$	δ_{12}	1.12	1.12	1.13	1.12	1.10	1.12
		δ_{23}	1.45	1.45	1.45	1.47	1.46	1.49
		δ_{34}	1.39	1.39	1.39	1.40	1.40	1.42
**Os**	(0001)	δ_{12}	2.06	2.06	2.16	2.08	2.05	2.10
		δ_{23}	2.14	2.14	2.06	2.17	2.13	2.19
		δ_{34}	2.18	2.18	2.24	2.21	2.17	2.23
	$(10\bar{1}0)$	δ_{12}	0.64	0.64	0.65	0.63	0.64	0.64
		δ_{23}	1.63	1.63	1.65	1.66	1.62	1.68
		δ_{34}	0.71	0.71	0.66	0.71	0.71	0.72
	$(11\bar{2}0)$	δ_{12}	1.25	1.25	1.26	1.26	1.23	1.28
		δ_{23}	1.35	1.35	1.35	1.37	1.36	1.38
		δ_{34}	1.38	1.38	1.41	1.39	1.36	1.42
**Ir**	(001)	δ_{12}	1.80	1.80	1.82	1.83	1.81	1.88
		δ_{23}	1.89	1.89	1.89	1.91	1.89	1.95
		δ_{34}	1.90	1.90	1.91	1.93	1.90	1.98
	(011)	δ_{12}	1.20	1.20	1.21	1.22	1.21	1.26
		δ_{23}	1.34	1.34	1.34	1.36	1.34	1.37
		δ_{34}	1.35	1.35	1.36	1.37	1.36	1.45
	(111)	δ_{12}	2.14	2.14	2.15	2.17	2.15	2.22
		δ_{23}	2.17	2.17	2.19	2.21	2.18	2.26

		δ_{34}	2.20	2.19	2.22	2.24	2.20	2.29
**Pt**	(001)	δ_{12}	1.90	1.90	1.91	1.92	1.86	1.99
		δ_{23}	1.94	1.94	1.96	1.96	1.92	2.02
		δ_{34}	1.95	1.95	1.98	1.97	1.94	2.02
	(011)	δ_{12}	1.20	1.20	1.21	1.20	1.13	1.24
		δ_{23}	1.50	1.50	1.50	1.53	1.53	1.57
		δ_{34}	1.34	1.34	1.35	1.36	1.29	1.42
	(111)	δ_{12}	2.29	2.29	2.30	2.32	2.27	2.41
		δ_{23}	2.24	2.24	2.25	2.27	2.21	2.34
		δ_{34}	2.24	2.24	2.25	2.27	2.22	2.34
**Au**	(001)	δ_{12}	2.00	2.00	2.01	2.03	2.03	2.15
		δ_{23}	2.03	2.03	2.05	2.06	2.06	2.17
		δ_{34}	2.03	2.03	2.05	2.06	2.06	2.18
	(011)	δ_{12}	1.25	1.25	1.27	1.25	1.25	1.28
		δ_{23}	1.56	1.56	1.57	1.61	1.60	1.74
		δ_{34}	1.37	1.37	1.39	1.37	1.37	1.41
	(111)	δ_{12}	2.36	2.36	2.37	2.41	2.40	2.55
		δ_{23}	2.34	2.34	2.36	2.39	2.37	2.50
		δ_{34}	2.34	2.34	2.35	2.38	2.37	2.48

Table. S8. Computed percentage of exposed surfaces for all the evaluated *xc* functionals, as a result of a Wulff shape evaluation.

TM	Surface	HL	PZ	AM05	revPBE	SCAN	BEEF
Sc	(0001)	32.48	32.48	32.15	33.62	30.89	33.14
	$(10\bar{1}0)$	41.58	41.47	40.13	40.70	45.47	32.46
	$(11\bar{2}0)$	25.94	26.05	27.72	25.68	23.64	34.40
Ti	(0001)	32.92	32.92	32.39	32.96	32.15	32.89
	$(10\bar{1}0)$	23.30	23.35	19.13	20.61	11.99	17.35
	$(11\bar{2}0)$	43.78	43.73	48.48	46.42	55.85	49.76
V	(0001)	26.09	26.07	29.64	28.77	29.03	30.24
	$(10\bar{1}0)$	65.67	65.59	61.24	63.91	64.48	57.00
	$(11\bar{2}0)$	8.24	8.35	9.12	7.32	6.49	12.76
Cr	(0001)	13.12	13.19	17.15	23.39	12.16	36.80
	$(10\bar{1}0)$	78.89	78.81	74.79	69.28	79.45	56.10
	$(11\bar{2}0)$	7.99	8.00	8.06	7.33	8.39	7.10
Fe	(0001)	16.01	36.65	46.59	24.41	17.29	28.93
	$(10\bar{1}0)$	78.19	5.05	0.00	67.15	60.24	61.96
	$(11\bar{2}0)$	5.80	58.30	53.41	8.44	22.47	9.11
Co	(001)	30.50	31.37	35.55	29.50	35.20	35.78
	(011)	69.50	68.63	52.44	0.00	55.98	51.01
	(111)	0.00	0.00	12.01	70.50	8.82	13.21
Ni	(001)	25.77	22.34	25.63	23.29	26.07	18.59
	(011)	11.14	0.00	8.87	6.48	11.11	0.00
	(111)	63.09	77.66	65.50	70.23	62.82	81.41
Cu	(001)	23.78	23.79	24.18	23.49	20.99	20.13
	(011)	9.91	9.92	8.99	8.12	5.67	2.61

	(111)	66.31	66.29	66.83	68.39	73.34	77.26
	(001)	39.18	39.33	51.48	42.27	47.43	77.04
**Zn**	(011)	60.82	60.67	12.44	57.73	52.57	22.96
	(111)	0.00	0.00	36.08	0.00	0.00	0.00
	(001)	33.35	33.35	33.24	34.15	31.85	35.13
**Y**	(011)	40.71	40.68	41.02	44.22	46.14	10.64
	(111)	25.94	25.98	25.74	21.64	22.01	54.23
	(001)	33.83	33.82	33.81	34.09	33.52	33.43
**Zr**	(011)	41.48	41.66	36.35	35.89	30.58	22.45
	(111)	24.69	24.52	29.84	30.02	35.90	44.12
	(0001)	12.96	12.88	14.73	15.52	16.60	14.94
**Nb**	$(10\bar{1}0)$	80.01	80.09	78.23	76.88	76.07	74.37
	$(11\bar{2}0)$	7.03	7.03	7.04	7.60	7.32	10.70
	(0001)	12.27	12.34	12.12	11.88	10.88	12.30
**Mo**	$(10\bar{1}0)$	82.72	82.64	83.11	82.92	83.29	82.48
	$(11\bar{2}0)$	5.01	5.02	4.77	5.20	5.83	5.23
	(001)	36.40	36.41	36.59	36.91	36.92	37.87
**Tc**	(011)	40.79	40.76	46.35	38.74	40.46	35.76
	(111)	22.80	22.83	17.05	24.35	22.62	26.37
	(0001)	36.14	36.14	36.32	36.10	36.14	36.29
**Ru**	$(10\bar{1}0)$	62.50	62.48	62.80	63.76	63.58	63.71
	$(11\bar{2}0)$	1.36	1.38	0.89	0.14	0.28	0.00
	(0001)	11.50	11.50	10.35	9.92	9.73	4.85
**Rh**	$(10\bar{1}0)$	0.00	0.00	0.00	0.00	0.00	0.00
	$(11\bar{2}0)$	88.50	88.50	89.65	90.08	90.27	95.15
	(0001)	15.04	13.17	11.46	13.30	8.68	5.05
**Pd**	$(10\bar{1}0)$	0.00	0.00	0.00	0.00	0.00	0.00
	$(11\bar{2}0)$	84.96	86.83	88.54	86.70	91.32	94.95
	(001)	23.64	23.65	23.18	21.42	24.74	11.70
**Ag**	(011)	7.67	7.69	4.45	1.46	11.22	0.00

	(111)	68.69	68.66	72.37	77.11	64.04	88.30
**Cd**	(001)	37.80	37.88	40.34	41.30	39.01	7.13
	(011)	62.20	62.12	59.66	58.70	60.99	92.87
	(111)	0.00	0.00	0.00	0.00	0.00	0.00
**Hf**	(001)	34.96	34.95	34.77	35.07	34.74	34.97
	(011)	36.53	36.55	32.79	32.21	33.19	29.63
	(111)	28.52	28.50	32.44	32.71	32.06	35.40
**Ta**	(001)	17.11	17.05	18.70	19.68	17.42	20.00
	(011)	78.74	78.82	77.92	77.09	78.13	76.46
	(111)	4.15	4.14	3.38	3.23	4.45	3.54
**W**	(001)	5.06	5.08	5.02	4.62	4.33	3.49
	(011)	88.55	88.55	88.59	87.22	90.36	87.41
	(111)	6.39	6.37	6.38	8.16	5.31	9.10
**Re**	(001)	36.03	36.02	36.11	36.33	36.17	37.27
	(011)	45.63	45.63	47.22	44.47	40.00	40.74
	(111)	18.34	18.34	16.67	19.20	23.83	21.99
**Os**	(001)	36.46	36.47	34.94	36.57	36.63	37.25
	(011)	63.54	63.53	65.06	63.43	63.37	62.75
	(111)	0.00	0.00	0.00	0.00	0.00	0.00
**Ir**	(001)	13.20	13.20	12.08	11.27	10.73	9.99
	(011)	0.00	0.00	0.00	0.00	0.00	0.00
	(111)	86.80	86.80	87.92	88.73	89.27	90.01
**Pt**	(0001)	18.25	18.24	16.51	16.74	13.68	7.29
	$(10\bar{1}0)$	0.00	0.00	0.00	0.00	0.00	0.00
	$(11\bar{2}0)$	81.75	81.76	83.49	83.26	86.32	92.71
**Au**	(0001)	14.93	14.94	12.96	12.82	13.38	0.22
	$(10\bar{1}0)$	0.00	0.00	0.00	0.00	0.00	0.00
	$(11\bar{2}0)$	85.07	85.06	87.04	87.18	86.62	99.78

References

(1) Janthon, P.; Luo, S.; Kozlov, S. M.; Viñes, F.; Limtrakul, J.; Truhlar, D. G.; Illas, F. Bulk Properties of Transition Metals: A Challenge for the Design of Universal Density Functionals. *J. Chem. Theory Comput.* **2014**, *10*, 3832-3839.

(2) Mills, K.C.; Su, Y. C. Review of surface tension data for metallic elements and alloys: Part 1 – Pure metals. *Int. Mater. Rev.* **2006**, *51*, 329.

(3) Helbert, B. M. The work function of the elements and its periodicity. *J. Appl. Phys.* **1997**, *48*, 4729.

(4) Vega, L.; Ruvireta, J.; Viñes, F.; Illas, F. Jacob's Ladder as Sketched by Escher. *J. Chem. Theory Comput.* **2018**, *14*, 395-403.
